# Differential effects of prophylactic iron supplementation on physiological gestational anemia and post-IDA gestational anemia: a study based on a rat model

**DOI:** 10.3389/fnut.2025.1650536

**Published:** 2025-09-12

**Authors:** Zelin Zhang, Limin Lai, Ziping Liu, Sili Liu, Liping Qu, Wenjun Zou

**Affiliations:** Chengdu University of Chinese Medicine, Chengdu, China

**Keywords:** pregnancy anemia, iron deficiency anemia, iron metabolism, iron supplements, fertility

## Abstract

**Objective:**

To observe the effects of iron supplementation on a physiological pregnancy and on pregnancy following iron deficiency anemia (IDA) caused by low-iron diet.

**Method:**

Physiological pregnancy anemia and IDA-induced pregnancy rat models were established, and the effects of preventive iron supplementation with ferrous succinate tablets (Sulifei, SLF) and polysaccharide–iron complex (Niferex, LFN) on pregnancy ability, embryonic development, anemia indicators, and iron content and metabolic indicators were observed in the model rats.

**Result:**

Anemia markers and body iron content were decreased in physiological pregnancy rat model, accompanied by abnormal oxidative stress and iron metabolism. In post-IDA gestational rat model, these markers were even more severely aggravated. SLF and LFN intervention improved body iron content, oxidative stress, and iron metabolism-related markers in physiological pregnant rats, but did not improve anemia-related markers. After 6 weeks of pretreatment with SLF and LFN, some reproductive toxicity effects were observed. SLF and LFN intervention in post-IDA gestational rat model improved anemia markers, body iron content, and iron metabolism-related markers. There were no significant differences in reproductive parameters between the two groups. Fetal weight and the average crown–rump length per litter increased in the LFN group.

**Conclusion:**

Post-IDA gestation further exacerbates iron deficiency anemia. Prophylactic iron supplementation can significantly improve physiological iron deficiency and iron metabolism during pregnancy but cannot improve iron deficiency anemia. In contrast, iron supplementation can significantly improve iron deficiency anemia in post-IDA gestation. To prevent or treat pregnancy complicated by IDA, iron supplementation is recommended either before the onset of IDA or after pregnancy.

## 1 Introduction

In the realm of global public health, the health of mothers and fetuses has always held a pivotal position. Especially during the pre-pregnancy and pregnancy periods, a woman’s health status not only directly relates to her own well-being but also serves as an indispensable core element for the healthy growth of the fetus (1). Among the numerous nutritional issues in pregnancy, IDA draws particular attention. Iron is involved in oxygen transport and energy metabolism processes and is a crucial HGB. Iron deficiency can lead to an insufficient supply of raw materials for HGB synthesis, thereby triggering IDA. According to available data, approximately 38% of pregnant women worldwide suffer from anemia placing the lives and health of millions of pregnant women and infants are at risk each year (2, 3). In particular, the risks of low birth weight and pre-term delivery associated with IDA are highly prominent. Iron is a vital constituent of HGB. Studies have shown that for every 10 g/L increase in a pregnant woman’s HGB level, the risk of maternal mortality decreases by 29%, and the risk of perinatal mortality simultaneously reduces by 28% (4). This dose–effect relationship indicates that optimizing iron nutritional status can lead to a leveraged decline in maternal and infant mortality rates.

Faced with this public health challenge, there are significant discrepancies in international prevention and control strategies. Countries such as the United States, the United Kingdom, and Latvia do not recommend routine iron supplementation for pregnant women (5, 6). Switzerland suggests conducting diagnostic tests for anemia (7) and measuring HGB and FER concentrations before initiating iron therapy. The World Health Organization recommends that all pregnant women routinely receive iron replacement therapy (8). Some researchers have also proposed (9) that iron supplementation for pregnant women should cover the entire reproductive cycle, from the pre-conception period to at least the end of lactation. In addition, many pregnant women self-administer iron-containing supplements without medical advice. However, the harsh reality is that despite the well-defined mechanisms of iron metabolism and clinical benefits, millions of pregnant women worldwide are still exposed to the risk of IDA. This contrast between theory and practice highlights significant scientific controversies regarding the implementation pathways and intervention timings of existing prevention and control strategies. Therefore, this study will address two key questions: First, whether preventive iron supplementation holds universal applicability; and second, how to define the optimal intervention window if supplementation is required.

In this study, female and male Sprague–Dawley (SD) rats were mated to allow pregnancy in females, thereby establishing a physiological gestational anemia model that simulates the physiological anemia observed during pregnancy in clinical settings. Meanwhile, we fed female rats a low-iron diet to induce IDA. After the development of IDA, these female rats were mated with male rats and allowed to become pregnant, simulating the gestational anemia state following IDA caused by poor nutritional status in clinical practice.

Two iron supplements were selected: ferrous succinate tablets (Sulifei, SLF) and polysaccharide–iron complex (Niferex, LFN). We conducted prophylactic administration of SLF and LFN to the physiological gestational anemia model and the post-IDA gestational rat model at different time points before pregnancy. We observed the effects of LFN and SLF on the conception capacity and embryonic development of rats with physiological gestational anemia and post-IDA gestational anemia, as well as on routine blood parameters, serum, and tissue iron contents.

We investigated the impact of prophylactic iron supplementation at different time points on gestational anemia and pregnancy outcomes. Additionally, we examined the effects on hepatic oxidative stress, serum–iron metabolism-related indicators, and the mRNA expression of tissue–iron metabolism-related indicators. From the perspectives of oxidative stress and iron metabolism, we initially explored the underlying mechanisms of action. Our aim is to provide a scientific basis for the rational selection of the timing of iron supplementation in clinical practice to prevent gestational IDA.

## 2 Materials and methods

### 2.1 Reagents and kits

SLF was manufactured by Nanjing Jinling Pharmaceutical Factory, Jinling Pharmaceutical Co., Ltd. (Batch No.: 171204), and LFN was produced by Kremers Urban Pharmaceuticals Inc., USA (Batch No.: 219095P1). The experimental diets included a standard AIN-93G diet (≈70 ppm Fe, Batch No.: D10012G) and a low-iron AIN-93G diet (<10 ppm Fe, Batch No.: RD18041204) purchased from Moldiets Co., Ltd.

#### 2.1.1 Experimental reagents

Organizational iron testing box: Nanjing Jiancheng Bioengineering Research Institute, Batch Number: 20190910; Serum Iron (SI) Test Kit: Nanjing Jiancheng Bioengineering Research Institute, Batch Number: 20190922; Serum Total Iron Binding Capacity (TIBC) Kit: Nanjing Jiancheng Bioengineering Institute, Batch Number: 20190922; Rat FE ELISA Kit: Wuhan Elirat Biotechnology Co., Ltd., Batch Number: ZB6ZDAJFG4; Superoxide Dismutase (SOD) Colorimetric Test Kit: Wuhan Eliruit Biotechnology Co., Ltd., Batch Number: CXJ4ZFT34; Total Antioxidant Capacity (T-AOC) Colorimetric Test Kit: Wuhan Eleruit Biotechnology Co., Ltd., Batch Number: TMF8LYICP9; Mouse GPX1 ELISA Kit: Wuhan Eli Lilly Biotechnology Co., Ltd., Batch Number: Y671YNY3EU; MDA ELISA Kit: Wuhan Eli Lilly Biotechnology Co., Ltd., Batch Number: P2Q66TW72K; Rat TF ELISA Kit: Wuhan Eli Lilly Biotechnology Co., Ltd., Batch Number: CXLWQ2DP14; Rat TFR1 ELISA Kit: Wuhan Elirat Biotechnology Co., Ltd., Batch Number: YVHZPUJM68; Rat EPO ELISA Kit: Wuhan Elirat Biotechnology Co., Ltd., Batch Number: 4E992JGCWT, etc.

#### 2.1.2 Experimental instruments

Fully automatic blood analyzer (five categories), manufactured by Hisense Medical Co., Ltd. in Japan, Model: XT-2000i; High-throughput tissue homogenizer, Beijing Dinghaoyuan Technology Co., Ltd., Model: V4800; Centrifuge, Thermo Scientific, Model: SorvallST16R; Multifunctional enzyme-linked immunosorbent assay (ELISA) reader, Themo Corporation, USA, Model: Varioska; Gene amplification instrument, Dongsheng Innovation Biotechnology Co., Ltd., Model: ETC811, etc.

### 2.2 Animals and diets

All animal experiments have been reviewed and approved by the Animal Care and Use Committee of Chengdu University of Traditional Chinese Medicine Affiliated Reproductive and Child Hospital, and conducted in accordance with the institution’s “Guidelines for the Care and Use of Experimental Animals.”

Specific pollution free (SPF) SD rats, including 7–8-week-old male and female rats, with the female rats being litter free, were purchased from Sibeifu (Beijing) Biotechnology Co., Ltd., Animal license number: SCXK (Beijing) 2019–0010. The experimental site is the Experimental Animal Room SYXK (Chuan) 2020–124, School of Pharmacy, Chengdu University of Traditional Chinese Medicine. All rats were fed according to the experimental requirements and maintained on a 12-h light/12-h dark cycle.

### 2.3 Experimental design

The experimental study was primarily divided into two parts: investigating the effects of drugs on rats in the physiological gestational anemia model and in the post-IDA gestational anemia model.

### 2.4 Physiological gestational anemia model

Normal SD rats were mated and allowed to become pregnant to simulate the physiological gestational anemia observed in clinical practice. The effects of prophylactic administration of the iron supplements, namely SLF and LFN, for 4 and 6 weeks were observed in the model rats. Specifically, after 5 days of adaptive feeding, female SD rats were weighed, and blood was collected from the orbital vein for a five-part differential complete blood count (CBC) test. Based on the principles of hemoglobin (HGB) levels and balanced body weight, the rats were divided into six groups: non-pregnant group (*n* = 8), physiological pregnancy group (*n* = 10), 4-week pre-treatment SLF group (*n* = 10), 6-week pre-treatment SLF group (*n* = 10), 4-week pre-treatment LFN group (*n* = 10), and 6-week pre-treatment LFN group (*n* = 10). The day of grouping was designated as D0.

For the drug-treated groups with 6-week and 4-week pre-treatments, SLF at a dose of 36 mg/kg and LFN at a dose of 27 mg/kg were administered by gavage on D1 and D15, respectively. Rats in the non-pregnant and physiological pregnancy groups were given an equal volume of purified water by gavage at a volume of 10 mL/kg once a day until the day of cesarean section of the female rats.

### 2.5 Post-IDA gestational anemia model

The IDA was induced in the animals by feeding them a purified low-iron diet for 7 weeks. After that, the rats were mated and allowed to become pregnant to replicate the post-IDA gestational anemia rat model. The effects of prophylactic administration of SLF and LFN on the model rats were then observed. After 5 days of adaptive feeding, female SD rats were weighed, and blood was collected from the orbital vein for a five-part differential CBC test. Based on the principles of HGB levels and balanced body weight, the rats were divided into five groups: non-pregnant group (*n* = 10), physiological pregnancy group (*n* = 14), post-IDA pregnancy group (*n* = 14), SLF group (*n* = 14), and LFN group (*n* = 14).

Rats in the SLF and LFN groups were given SLF at a dose of 36 mg/kg and LFN at a dose of 27 mg/kg by gavage on D1, respectively. Rats in the other groups were given an equal volume of purified water by gavage at a volume of 10 mL/kg once a day. On D1, female rats in the non-pregnant and physiological pregnancy groups were fed a standard formula diet (≈70 ppm Fe), while female rats in the other groups were fed a low-iron diet (<10 ppm Fe) until the day of cesarean section.

Except for the non-pregnant group, in both the physiological gestational anemia model and the post-IDA gestational anemia model, female rats were cohoused with male rats (2 females and 1 male per cage) on D42 and D49, respectively. The male rats were removed 24 h later, and cohousing for mating continued for 14 consecutive days. Vaginal plug examination was used to confirm pregnancy. The day of pregnancy detection was designated as gestational day (GD) 0, the next day as GD 1, and so on to estimate the gestational age. Cesarean sections were performed on GD 20. Blood was collected from the abdominal aorta; a portion was immediately tested for anemia-related indicators, and a portion was centrifuged and separated from serum before being frozen at −80°C. After blood collection, the rats were euthanized. The liver, small intestine, and placenta of the female rats were collected and stored at −80 °C for future use.

### 2.6 Observation of rat fertility and embryonic development

On GD 20, the fetuses were removed. The weights of the uterine contents (including fetuses) and the uterus were measured. The number of corpora lutea, live fetuses, early resorptions, late resorptions, absorbed fetuses, and implantation sites were counted. Routine examination for malformations was conducted. The amniotic fluid and placenta of the fetuses were visually inspected. The placenta was separated, and the body weight, placental weight, and crown–rump length of the fetuses were measured. The number of pregnant animals and the conception rate were calculated.

### 2.7 Detection of CBC parameters, and serum and tissue iron content

CBC parameters were measured using a blood analyzer, including the determination of RBC, HGB level, and HCT in peripheral blood.

Serum iron-related parameters: A serum iron (SI) kit, a total iron-binding capacity (TIBC) kit, and a ferritin (FE) kit were employed. The levels of SI, TIBC, and FE in the serum of female rats were detected according to the instructions provided in the kits. Transferrin saturation (TSAT) was then calculated using the formula: TSAT (%) = (SI/TIBC) × 100%.

Tissue iron content: A tissue iron kit was used to measure the iron content in the liver, small intestine, and placenta tissues of female rats, following the kit instructions.

### 2.8 Detection of oxidative stress-related indicators

The levels of superoxide dismutase (SOD), total antioxidant capacity (T-AOC), glutathione peroxidase-1 (GPX1), and malondialdehyde (MDA) in the liver were measured using relevant ELISA kits, including those for SOD, T-AOC, GPX1, and MDA. The measurements were carried out according to the instructions provided with the kits.

### 2.9 ELISA-based detection of serum iron metabolism-related indicators

Whole blood was collected, and serum was separated by cryogenic centrifugation at 3000 r/min at 4 °C. Subsequently, ELISA kits for serum transferrin (TF), serum transferrin receptor 1 (TFR1), and erythropoietin (EPO) were used to detect the levels of TF, TFR1, and EPO in the serum, following the kit instructions.

### 2.10 Determination of mRNA expression of tissue iron metabolism-related indicators

Appropriate portions of rat liver, small intestine, and placental tissues were collected and placed in 1000 μL of TRIzol. RNA was extracted according to the manufacturer’s instructions, and the purity and integrity of the RNA were assessed using agarose gel electrophoresis.

Reverse transcription was performed using the PrimeScript™ II 1st Strand cDNA Synthesis Kit (Takara Bio Inc.) to obtain cDNA.

For the amplification setup, the 2 × HotStart Power Taq PCR StarMix with loading dye was utilized. The reaction mixture consisted of 1 μL each of DNA template, forward primer, and reverse primer (primer sequences are shown in Table 1), 10 μL of 2 × HotStart Power Taq PCR StarMix, and ddH_2_O to a total volume of 20 μL.

**Table 1:** Primer sequence.

Primers	Primer sequence 5′–3′
GAPDH-F	5’-AGTGCCAGCCTCGTCTCATA-3’
GAPDH-R	5’-ACCAGCTTCCCATTCTCAGC-3’
FPN1-F	5’-CCAACCCGCTTCCATAAGGC-3’
FPN1-R	5’-CCGAAAGACCCCAAAGGACA-3’
TFRI-F	5’-GGAACCAGACCGCTACAT-3’
TFRI-R	5’-TCAATCGGATGCTTTACG-3’
HAMP-F	5’-GCTGCCTGTCTCCTGCTT-3’
HAMP-R	5′-GGTGTCTCGCTTCCTTCG-3’
DMTI-F	5’-AGTAAACACTGGGTCAGCCT-3’
DMTI-R	5’-ACGGCACATACTTGTGGCTA-3’
DMT + IRE-F	5’-GCCTGTCTGTCTGTCTTTGC-3’
DMT + IRE-R	5’-CCCAGTGTTTCCCAACTAACA-3’
DMT-IRE-F	5’-TAGATGACCAACAGCCCAGA-3’
DMT-IRE-R	5’-CACAGCCGTTAGCTTTACCC-3

The amplification conditions were as follows: initial denaturation at 95°C for 10 min, followed by 32 cycles of denaturation at 95°C for 30 s, annealing at 60°C for 30 s, and extension at 72°C for 30 s.

After cooling on ice, 10 μL of the amplified mRNA was subjected to 1.0% agarose gel electrophoresis. The gel was then imaged and photographed using an ImageQuant LAS 500 integrated imaging system. The grayscale values of the target bands were analyzed using Image J software, and the ratio of the grayscale value of the target gene to that of the internal reference gene was calculated.

### 2.11 Statistical methods

The experimental data were presented as mean ± standard error (x̄±S). Statistical analyses were conducted using SPSS 21.0. For normally distributed data, one-way analysis of variance (ANOVA) was applied, while for non-normally distributed data, non-parametric tests were utilized. A statistically significant difference between datasets was indicated when *p* < 0.05.

## 3 Experimental results

### 3.1 Effects of iron supplements on the fertility of rats with gestational anemia

**Figure 1:**
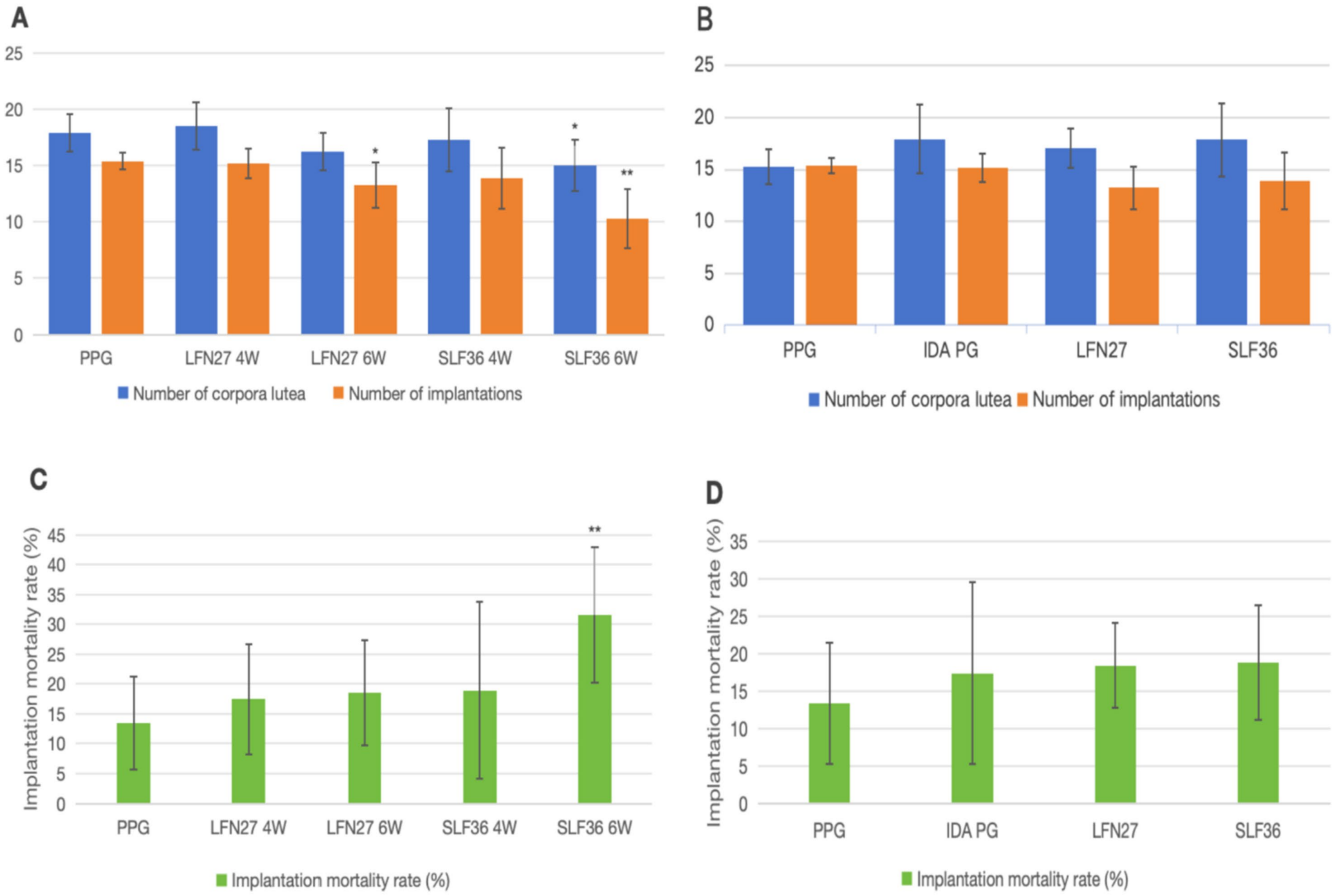
Effects of iron supplements on the fertility of pregnant anemia rats. **(A, C)** Physiological pregnancy anemia model. **(B, D)** Pregnancy anemia model after IDA. Compared with the physiological pregnancy group, *P < 0.05; **P < 0.01.

In the physiological gestational anemia model, as shown in Figure 1, there were no significant differences in the pregnancy outcomes among the groups of rats that were caged together for mating within 2 weeks starting from D42 (*p* > 0.05). In this study, the fertility of rats was assessed by the number of corpora lutea, the number of implantation sites, and the implantation mortality rate.

Compared to the physiological pregnancy group, the number of implantation sites in the LFN and SLF groups of the 6-week pre-administration physiological pregnancy group was significantly reduced (*p* < 0.05 or *p* < 0.01). Additionally, the number of corpora lutea in the SLF group was also significantly decreased (*p* < 0.05), and the implantation mortality rate in the SLF group was significantly increased (*p* < 0.01).

In the post-IDA gestational anemia model, rats in each group were caged together for mating within 2 weeks starting from D49 (when anemia occurred in the IDA pregnancy group). Compared to the IDA pregnancy group, the number of pregnant rats in the SLF group decreased (*p* < 0.05), while there were no statistically significant differences in other groups (*p* > 0.05). There were no significant differences in the number of corpora lutea, the number of implantation sites, and the implantation mortality rate among all groups (*p* > 0.05).

### 3.2 Effects of iron supplements on embryonic development in rats with gestational anemia embryo count

**Figure 2:**
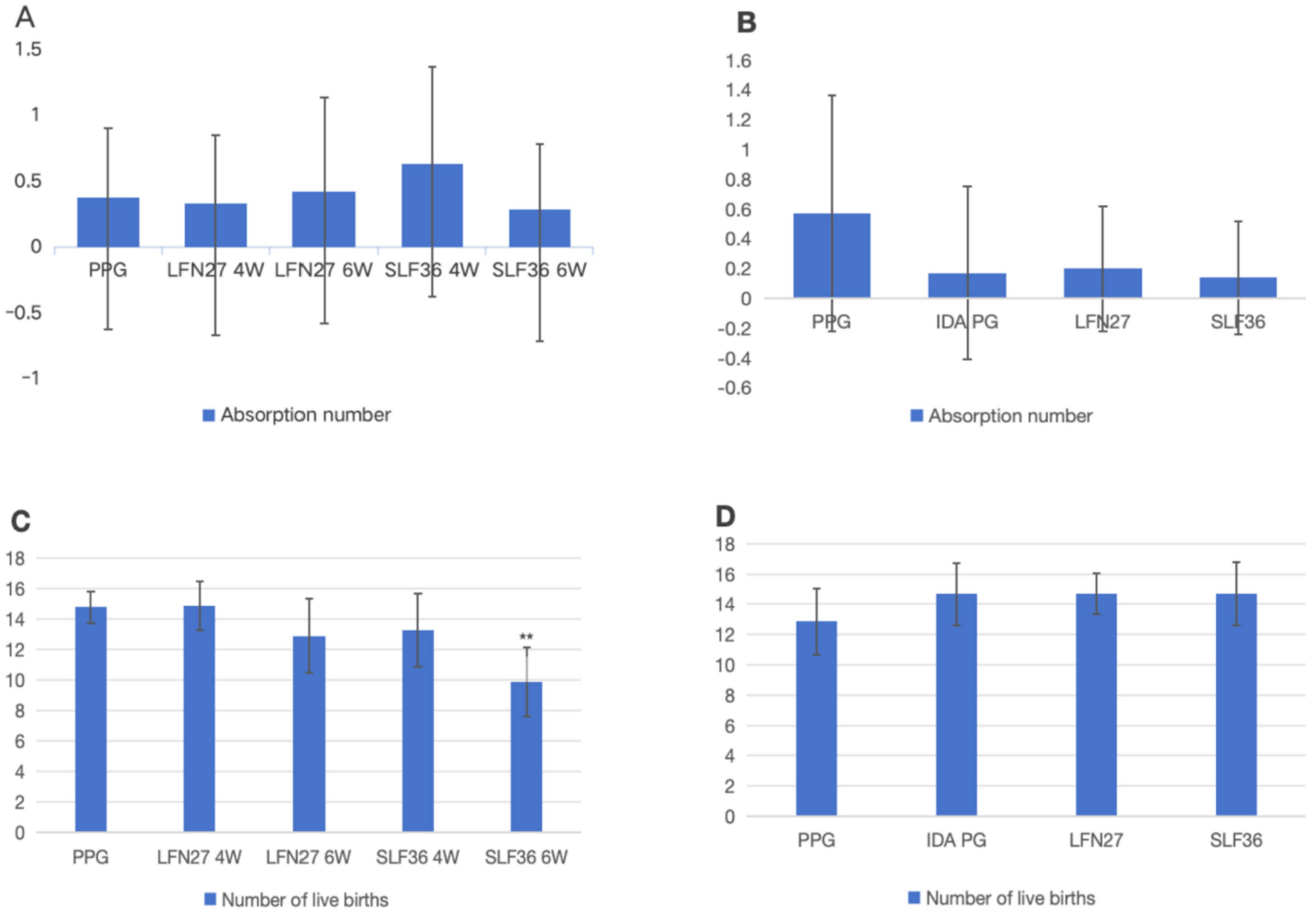
Effect of iron supplements on the number of embryos in pregnant anemic rats. **(A, C)** Physiological pregnancy anemia model. **(B, D)** Pregnancy anemia model after IDA. Compared with the physiological pregnancy group, **P < 0.01.

As shown in Figure 2, when compared with the physiological pregnancy group, the number of live fetuses in the 6-week pre-administration SLF group decreased significantly (*p* < 0.01). However, there were no significant differences in the number of resorbed fetuses, early fetal deaths, and late fetal deaths among the groups (*p* > 0.05). In the post-IDA gestational anemia model, there were no statistically significant differences in the aforementioned indicators across all the groups (*p* > 0.05).

### 3.3 Embryonic and litter development

**Figure 3:**
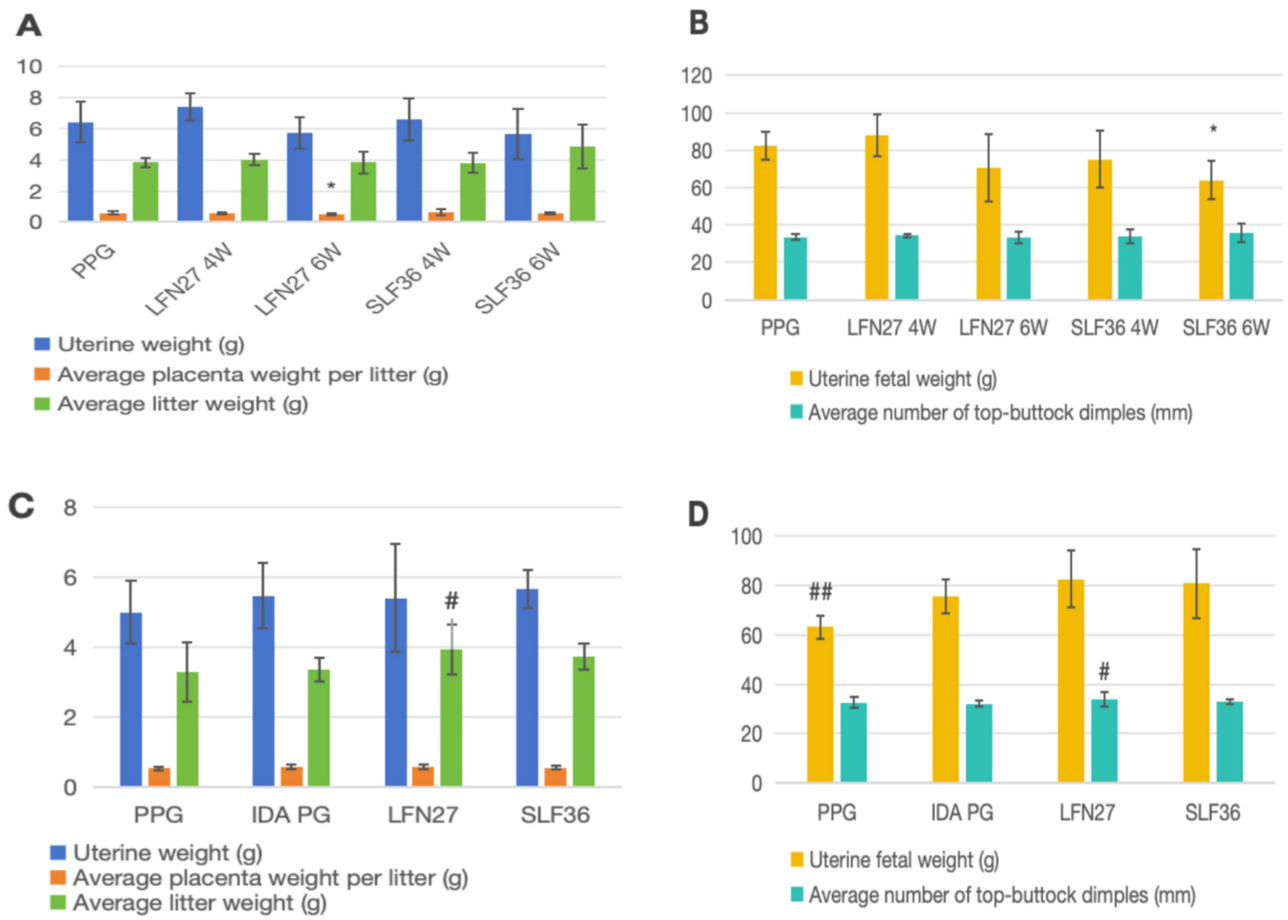
Effects of iron supplements on embryo and offspring development in pregnant anemia rats. **(A, B)** Physiological pregnancy anemia model. **(C, D)** Pregnancy anemia model after IDA. Compared with the physiological pregnancy group, *P < 0.05; compared with the IDA pregnancy group, ^#^*P* < 0.05; ^##^*P* < 0.01.

As shown in Figure 3, compared to the physiological pregnancy group, the mean placental weight per litter in the 6-week pre-administration LFN group was significantly reduced (*p* < 0.05), and the mean fetal–uterine weight in the six-week pre-administration SLF group was markedly decreased (*p* < 0.05). No other significant differences were observed (*p* > 0.05). In the post-IDA gestational anemia model, there were no notable differences in any of the indicators between the IDA-pregnant rats and the normal pregnancy group (*p* > 0.05). After 6 weeks of pre-administration of LFN, the mean fetal body weight per litter and the mean crown–rump length per litter in the post-IDA-pregnant rats increased significantly (*p* < 0.05).

### 3.4 Effects of iron supplements on routine blood parameters in rats with gestational anemia

**Figure 4:**
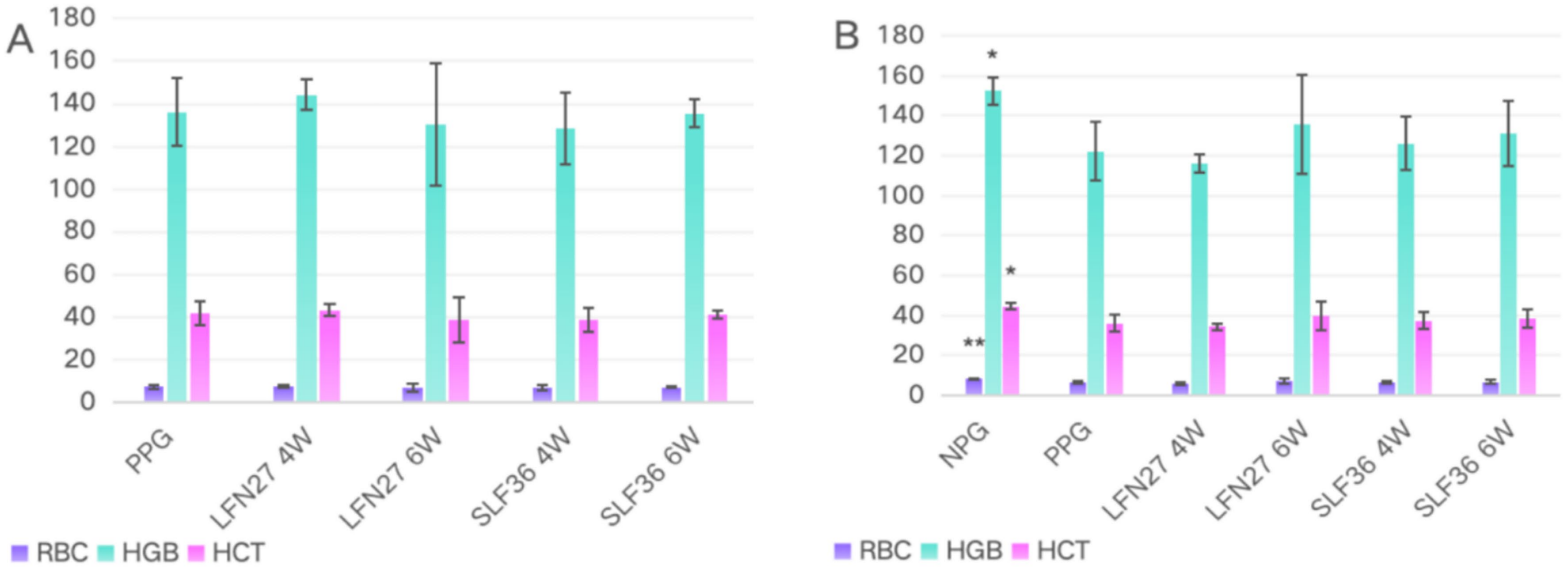
Blood routine of physiological pregnancy anemia rats treated with iron supplements. **(A)** Physiological pregnancy anemia model (D42) and **(B)** physiological pregnancy anemia model (GD20). Compared with the physiological pregnancy group, *P < 0.05, **P < 0.01. Compared to the IDA pregnancy group, ^#^*P* < 0.05.

In the physiological gestational anemia rat model, as shown in Figure 4, there were no significant differences in the relevant routine blood parameters among the groups after 4 and 6 weeks of consecutive intragastric administration of the drugs before mating (D42) (*p* > 0.05). On GD20, compared to the non-pregnant group, the serum levels of RBC, HGB, and HCT in the physiological pregnancy group were significantly decreased (*p* < 0.05 or *p* < 0.01), indicating the onset of physiological gestational anemia. In comparison to the physiological pregnancy group, neither 4-week nor 6-week pre-administration of LFN or SLF could significantly reverse the changes in RBC, HGB, and HCT (*p* > 0.05).

**Figure 5:**
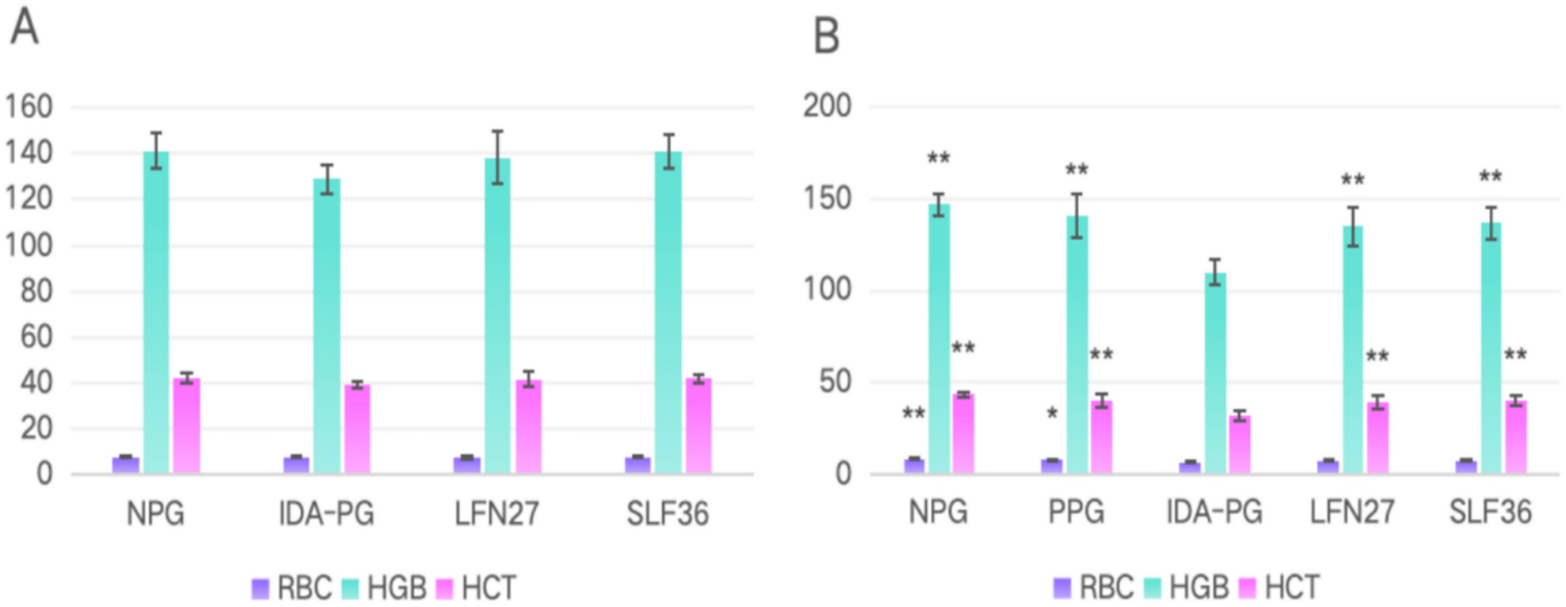
Effects of iron supplements on blood routine of IDA pregnant rats. **(A)** Pregnancy anemia model after IDA (D49) and **(B)** pregnancy anemia model after IDA (GD20). Compared to the IDA pregnancy group, *P < 0.05, **P < 0.01; a, merge data from non-pregnant group and physiological pregnant group.

In the post-IDA gestational anemia rat model, as shown in Figure 5, 7 weeks of consecutive low-iron diet feeding before mating (D49) resulted in a significant decrease in both HGB and HCT levels in rats (*p* < 0.01), leading to an anemic state. Concurrent administration of SLF during this period could significantly reverse the changes in HGB and HCT (*p* < 0.05 or *p* < 0.01), while the LFN group showed a certain trend of improvement. On GD20, all the parameters in the IDA-pregnant group further declined compared to the pre-pregnancy state. The levels of RBC, HGB, and HCT were not only significantly lower than those in the non-pregnant group (*p* < 0.01) but also markedly lower than those in the physiological pregnancy group (*p* < 0.05 or *p* < 0.01). Compared to the IDA-pregnant group, continuous administration of LFN and SLF until GD20 significantly improved the levels of HGB and HCT in rats (*p* < 0.05 or *p* < 0.01).

### 3.5 Effects of iron supplements on serum iron-related indicators and tissue iron content in pregnant rats

**Figure 6:**
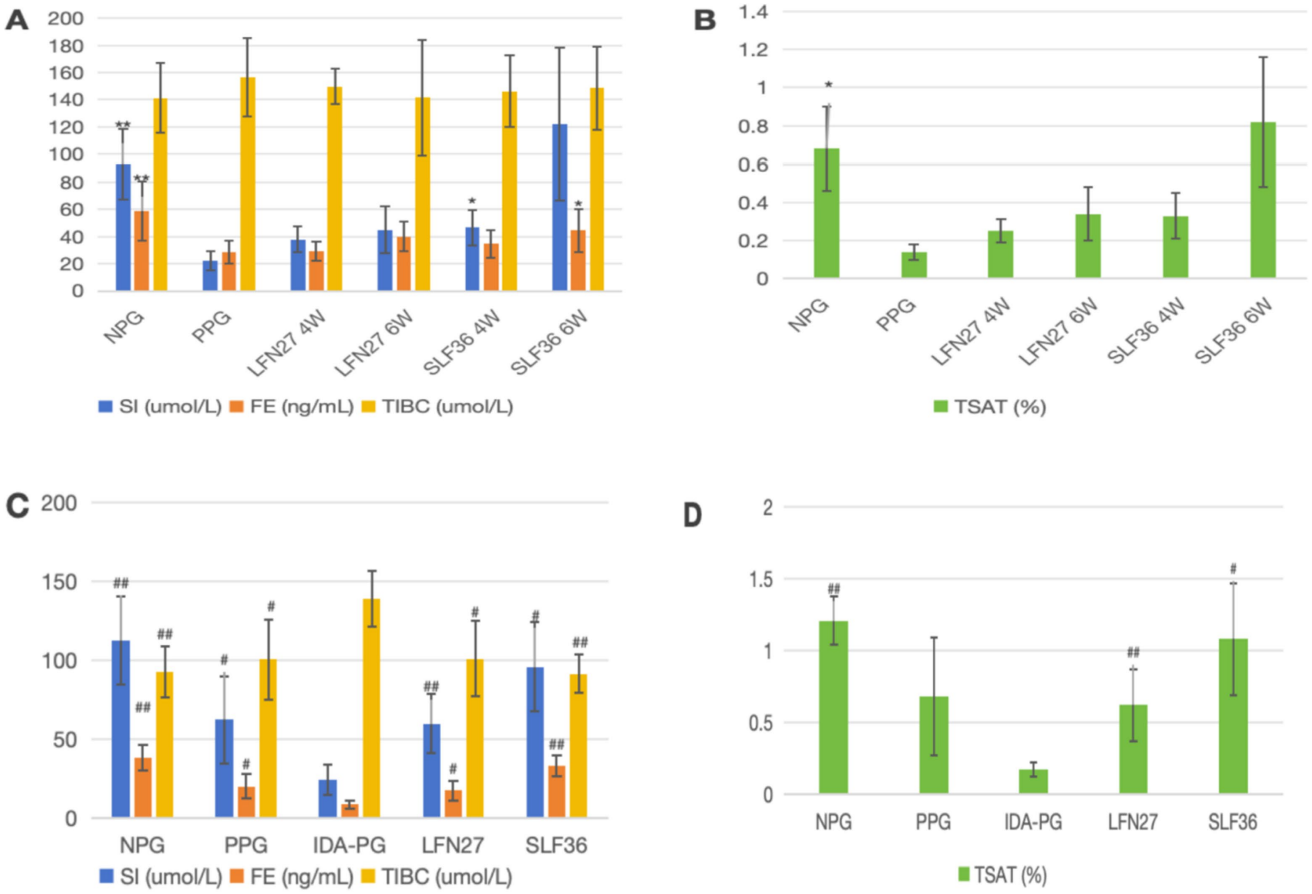
Effects of GD20 iron supplement on serum iron-related indicators in pregnant anemia rats. **(A, B)** Physiological pregnancy anemia model. **(C, D)** Pregnancy anemia model after IDA. Compared with the physiological pregnancy group, *P < 0.05; **P < 0.01. Compared with the IDA pregnancy group, ^#^*P* < 0.05; ^##^*P* < 0.01.

**Figure 7:**
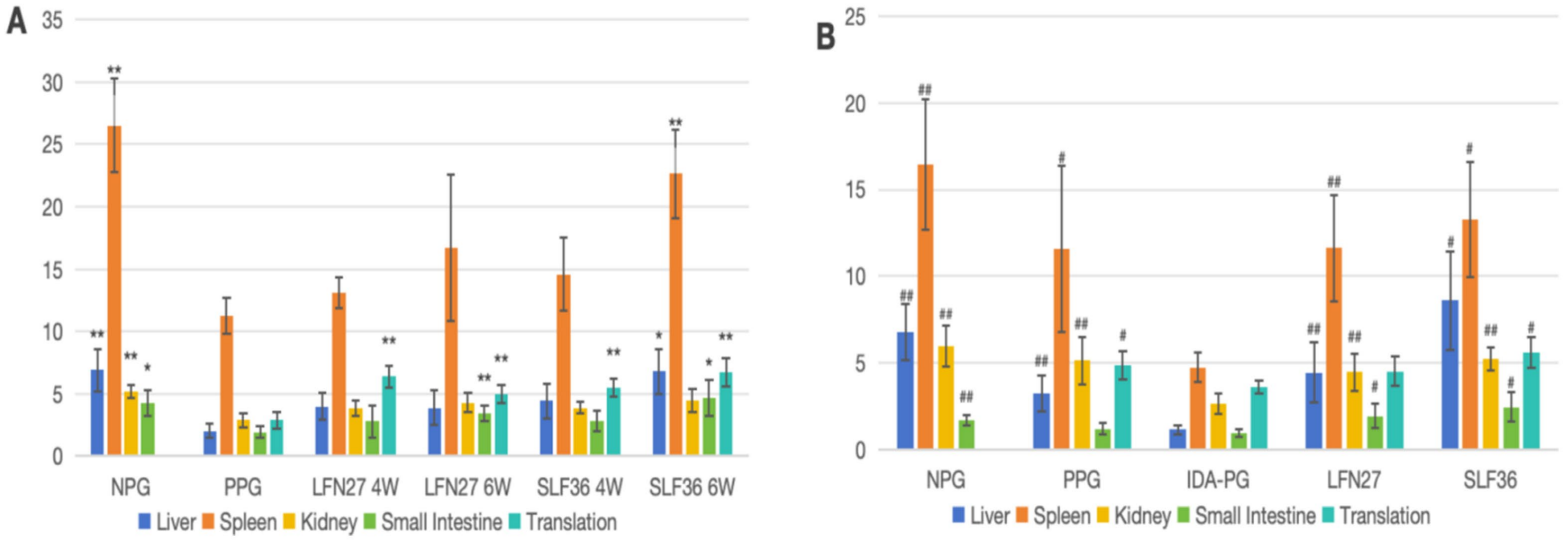
Effect of GD20 iron supplement on tissue iron content in pregnant anemic rats. **(A)** Physiological pregnancy anemia model. **(B)** Pregnancy anemia model after IDA. Compared with the physiological pregnancy group, *P < 0.05; **P < 0.01. Compared with the IDA pregnancy group, ^#^*P* < 0.05; ^##^*P* < 0.01.

In the physiological gestational anemia rat model, as demonstrated in Figures 6, 7, compared to the non-pregnant group, the serum levels of SI, FE, and TSAT in the physiological pregnancy group were significantly decreased (*p* < 0.05 or *p* < 0.01). There was a certain increasing trend in TIBC. The iron content in the liver, spleen, kidney, and small intestine was also markedly reduced (*p* < 0.05 or *p* < 0.01). These findings suggest that normal rats develop a state of physiological iron deficiency after pregnancy.

When compared with the physiological pregnancy group, pre-administration of SLF increased the serum levels of SI and FE to varying degrees (*p* < 0.05), and the LFN group showed a tendency toward an increase. The iron content in various organs of the animals in the SLF and LFN groups increased to different extents. Specifically, in the six-week pre-administration SLF group, the iron content in the liver, spleen, and small intestine was significantly elevated (*p* < 0.05 or *p* < 0.01). In the six-week pre-administration LFN group, the iron content in the small intestine was significantly increased (*p* < 0.01). Additionally, the iron content in the placenta was significantly higher in both the LFN and SLF groups (*p* < 0.05 or *p* < 0.01).

**Figure 8:**
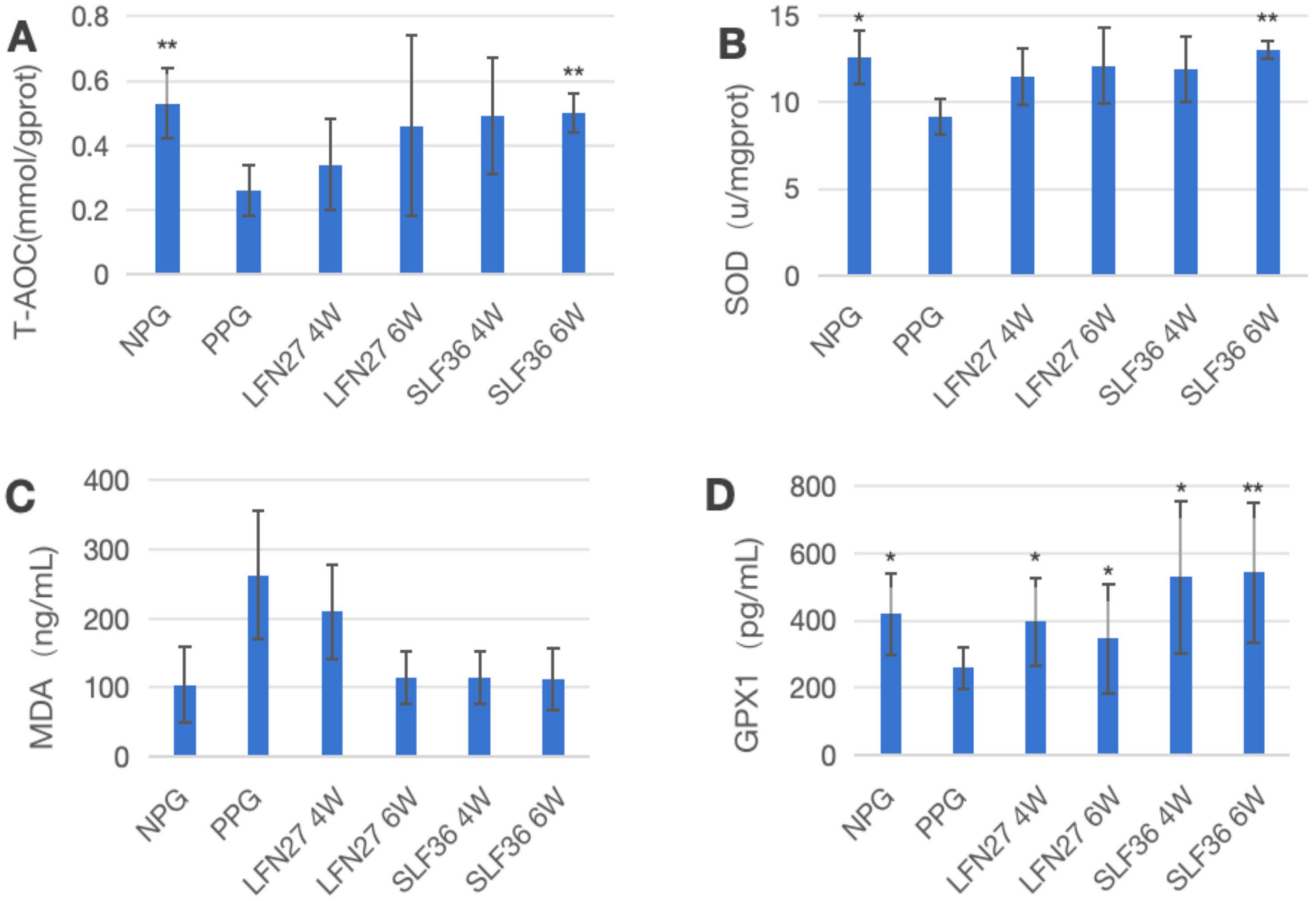
Effects of GD20 iron supplement on liver oxidative stress related indicators in physiological pregnancy group. **(A)** T-AOC; **(B)** SOD; (C) MDA; (D) GPX1. Compared with the physiological pregnancy group, *P < 0.05; **P < 0.01.

In the post-IDA gestational anemia rat model, as shown in Figures 7, 8, feeding a low-iron diet exacerbated the changes in all indicators in the IDA-pregnant group. There were significant differences compared to the non-pregnant group (*p* < 0.01). The levels of SI and FE were significantly lower than those in the physiological pregnancy group (*p* < 0.05), while TIBC was significantly higher (*p* < 0.05). Although there was a decreasing trend in TSAT (*p* > 0.05), the iron content in the liver, spleen, kidney, and placenta was markedly lower than that in the physiological pregnancy group (*p* < 0.05 or *p* < 0.01).

Compared to the IDA-pregnant group, the SI, FE, TIBC, and TSAT levels in the SLF and LFN groups were significantly increased (*p* < 0.05 or *p* < 0.01). The iron content in the liver, spleen, kidney, and small intestine was also significantly elevated (*p* < 0.05 or *p* < 0.01). In the SLF group, the placental iron content was significantly increased (*p* < 0.05). Moreover, there was a tendency for the liver iron level in the SLF group to be higher than that in the normal group.

### 3.6 Effects of iron supplements on oxidative stress-related indicators in pregnant rats

In the physiological gestational anemia rat model, as shown in Figure 8, compared to the non-pregnant group, the levels of T-AOC, SOD, and GPX1 in the liver of the physiological gestational anemia model group were significantly decreased (*p* < 0.05 or *p* < 0.01). There was a certain increasing trend in MDA levels.

When compared to the physiological pregnancy group, the six-week pre-administration SLF group showed a significant increase in liver T-AOC and SOD levels (*p* < 0.01). Both the four-week and six-week pre-administration LFN and SLF groups showed a significant elevation in liver GPX1 levels (*p* < 0.05 or *p* < 0.01). Additionally, there was a decreasing trend in liver MDA levels in both the LFN and SLF groups, although the differences were not statistically significant (*p* > 0.05).

**Figure 9:**
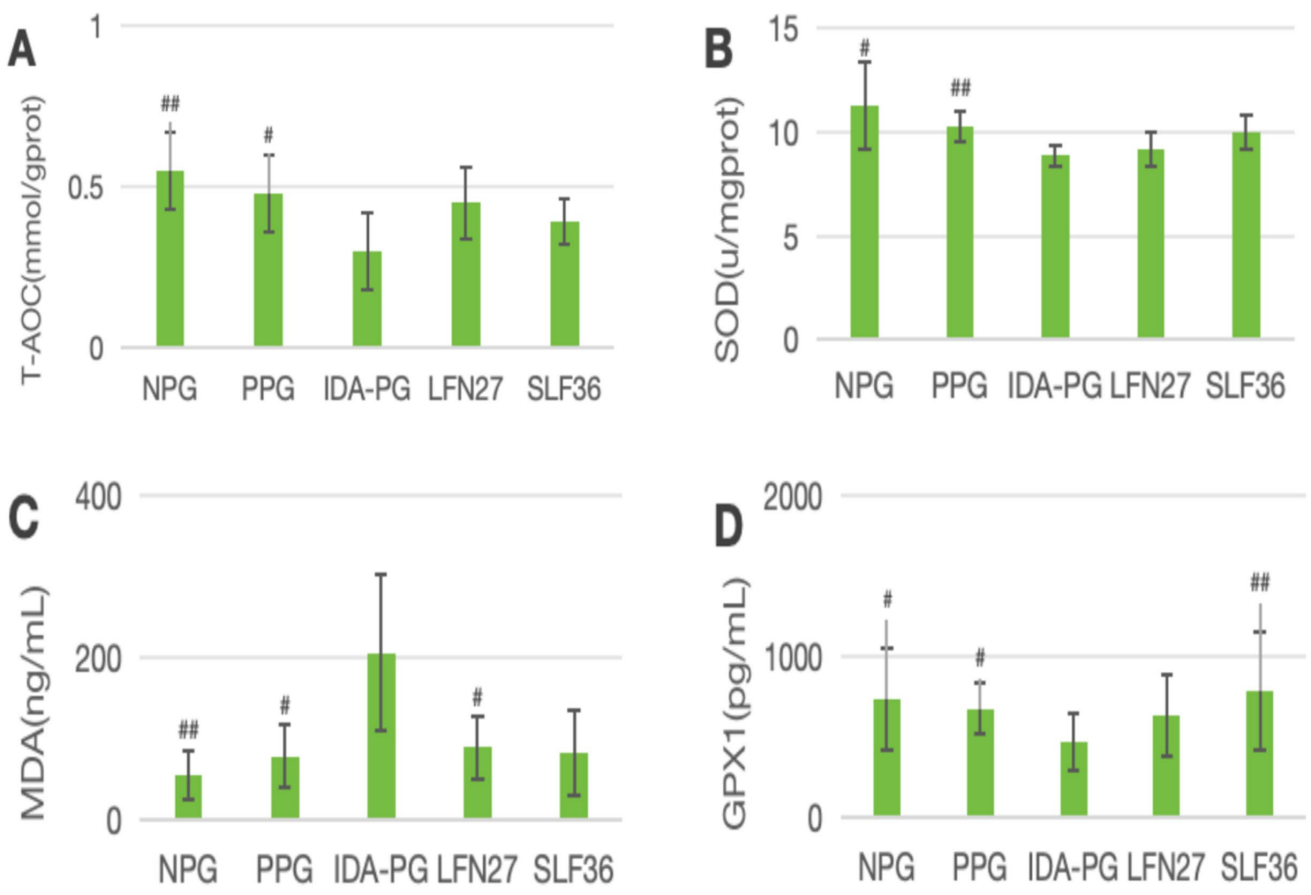
Effects of GD20 iron supplement on liver oxidative stress related indicators in pregnancy anemia model after IDA. **(A)** T-AOC; **(B)** SOD; **(C)** MDA; **(D)** GPX1. Compared with the IDA pregnancy group, ^#^*P* < 0.05; ^##^*P* < 0.01.

In the post-IDA gestational anemia rat model, as shown in Figure 9, feeding a low-iron diet exacerbated the changes in all indicators in the IDA-pregnant group. The levels of T-AOC, SOD, and GPX1 in the IDA-pregnant group were significantly lower than those in both the physiological pregnancy group and the non-pregnant group (*p* < 0.05 or *p* < 0.01). In contrast, the MDA level was significantly higher than those in the physiological pregnancy group and the non-pregnant group (*p* < 0.05).

Compared to the IDA-pregnant group, the LFN group showed a significant increase in MDA levels (*p* < 0.05), while the SLF group displayed a non-significant increase in MDA levels (*p* > 0.05). Conversely, the SLF group had a significantly higher GPX1 level (*p* < 0.05), and the LFN group showed a non-significant increase in GPX1 levels (*p* > 0.05).

### 3.7 Effects of iron supplements on serum iron metabolism-related indicators in pregnant rats

**Figure 10:**
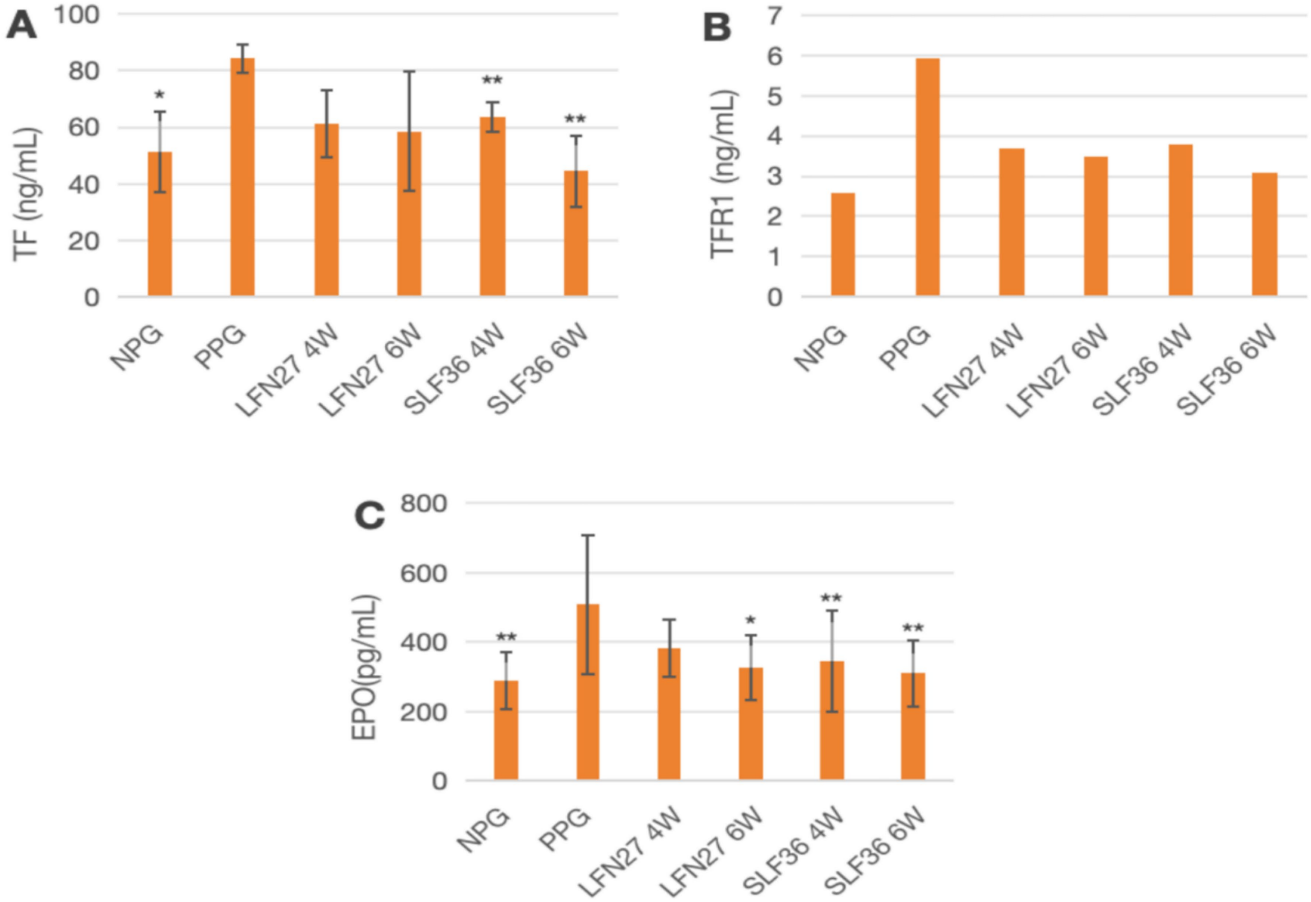
Effects of LFN and SLF on serum iron metabolism-related indicators in Physiological pregnancy anemia model. Compared to the physiological pregnancy group, *P < 0.05, **P < 0.01. Compared to the IDA pregnancy group, ^#^*P* < 0.05. **(A)** TF; **(B)** TFR1; **(C)** EPO.

**Figure 11:**
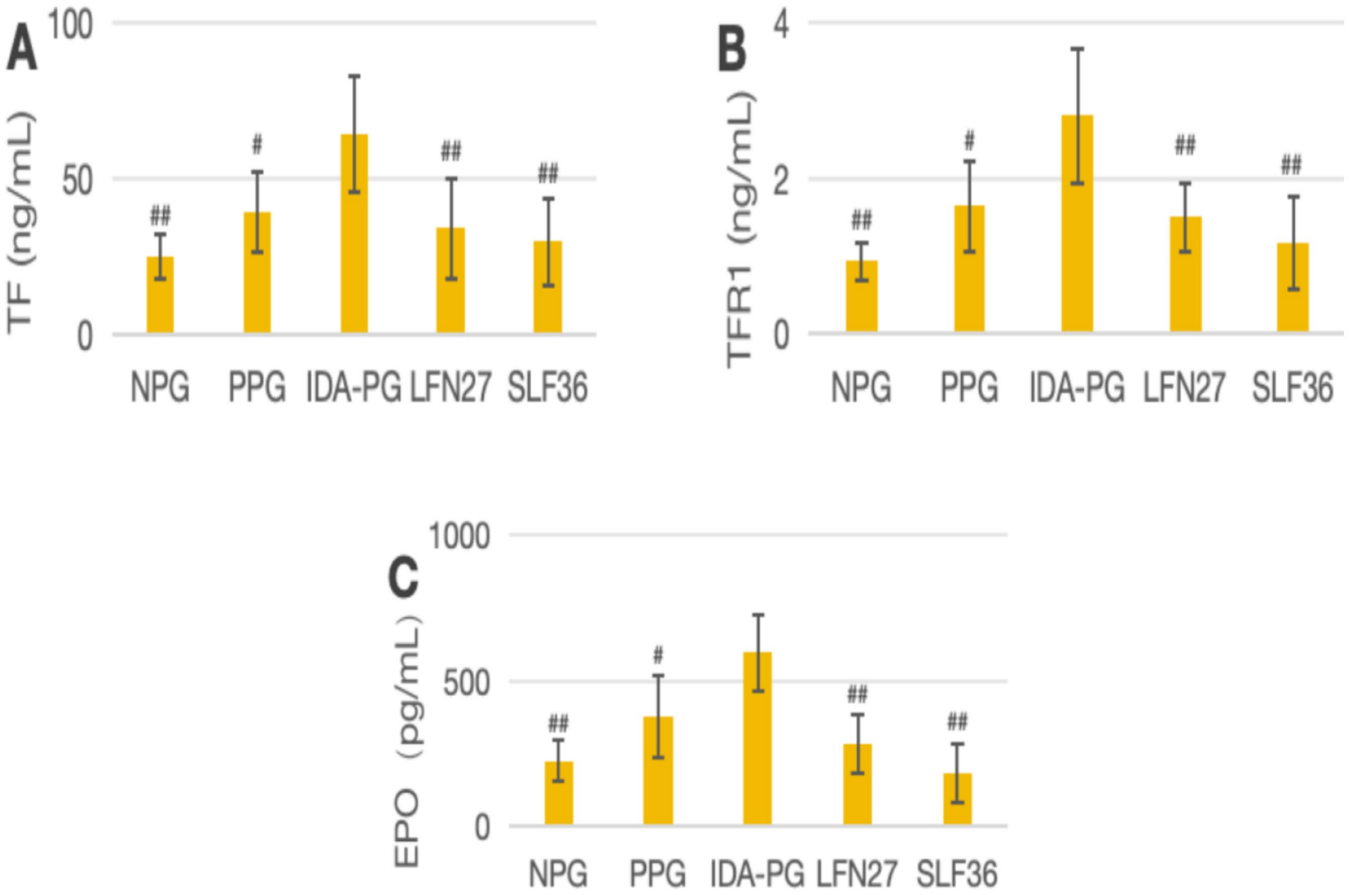
Effects of LFN and SLF on serum iron metabolism-related indicators in pregnancy anemia model after IDA. **(A)** TF; **(B)** TFR1; **(C)** EPO. Compared with the IDA pregnancy group, ^#^*P* < 0.05; ^##^*P* < 0.01.

In the physiological gestational anemia rat model, as demonstrated in Figures 10, 11, when compared to the non-pregnant group, the levels of TF and EPO in the physiological pregnancy group were significantly elevated (*p* < 0.05 or *p* < 0.01). There was a certain increasing trend in TFR1 levels.

When compared to the physiological pregnancy group, the LFN and SLF groups showed varying degrees of reversal trends in TF, EPO, and TFR1 levels. Specifically, in the four-week and six-week pre-administration SLF groups, the TF and EPO levels were significantly decreased (*p* < 0.05 or *p* < 0.01). In the six-week pre-administration LFN group, the EPO level was markedly reduced (*p* < 0.05).

In the post-IDA gestational anemia rat model, as shown in Figure 10, feeding a low-iron diet exacerbated the changes in all indicators in the IDA-pregnant group. The levels of TF, TFR1, and EPO in the post-IDA-pregnant group were significantly higher than those in the non-pregnant group (*p* < 0.01) and the physiological pregnancy group (*p* < 0.05). Compared to the IDA-pregnant group, the SLF and LFN groups exhibited significant decreases in serum TF, TFR1, and EPO levels (*p* < 0.05 or *p* < 0.01).

### 3.8 Effects of iron supplements on mRNA expression of tissue iron metabolism-related indicators in pregnant rats

**Figure 12:**
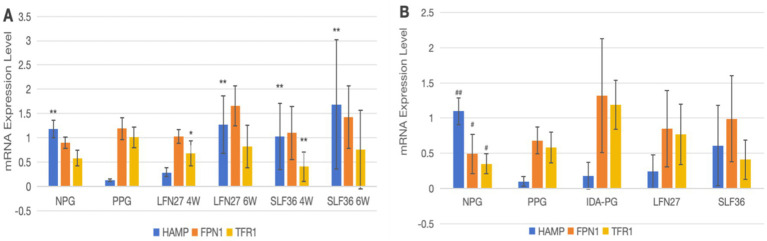
Effects of iron supplements on mRNA expression of liver iron metabolism-related indicators in pregnant anemic rats. **(A)** Physiological pregnancy anemia model; **(B)** pregnancy anemia model after IDA. Compared with the physiological pregnancy group, *P < 0.05; **P < 0.01; compared with the IDA pregnancy group, ^#^*P* < 0.05; ^##^*P* < 0.01.

As shown in Figure 12, regarding mRNA expression of liver iron metabolism-related indicators, when compared to the non-pregnant group, the physiological pregnancy group exhibited a significant decrease in HAMP mRNA levels (*p* < 0.01). There was an increasing trend in TFR1 mRNA levels, while no obvious change was observed in FPN1 mRNA levels.

When compared to the physiological pregnancy group, the six-week pre-administration LFN group, as well as the four-week and six-week pre-administration SLF groups, showed a significant increase in HAMP mRNA levels (*p* < 0.01). Feeding a low-iron diet to induce IDA in pregnant rats further exacerbated the changes in all indicators. Compared to the non-pregnant group, statistically significant differences were observed in all these indicators (*p* < 0.05 or *p* < 0.01).

When compared to the IDA-pregnant group, there was no significant difference in liver HAMP mRNA levels between the LFN and SLF groups. There was a certain reversal trend in FPN1 and TFR1 mRNA levels, although the differences were not statistically significant (*p* > 0.05).

**Figure 13:**
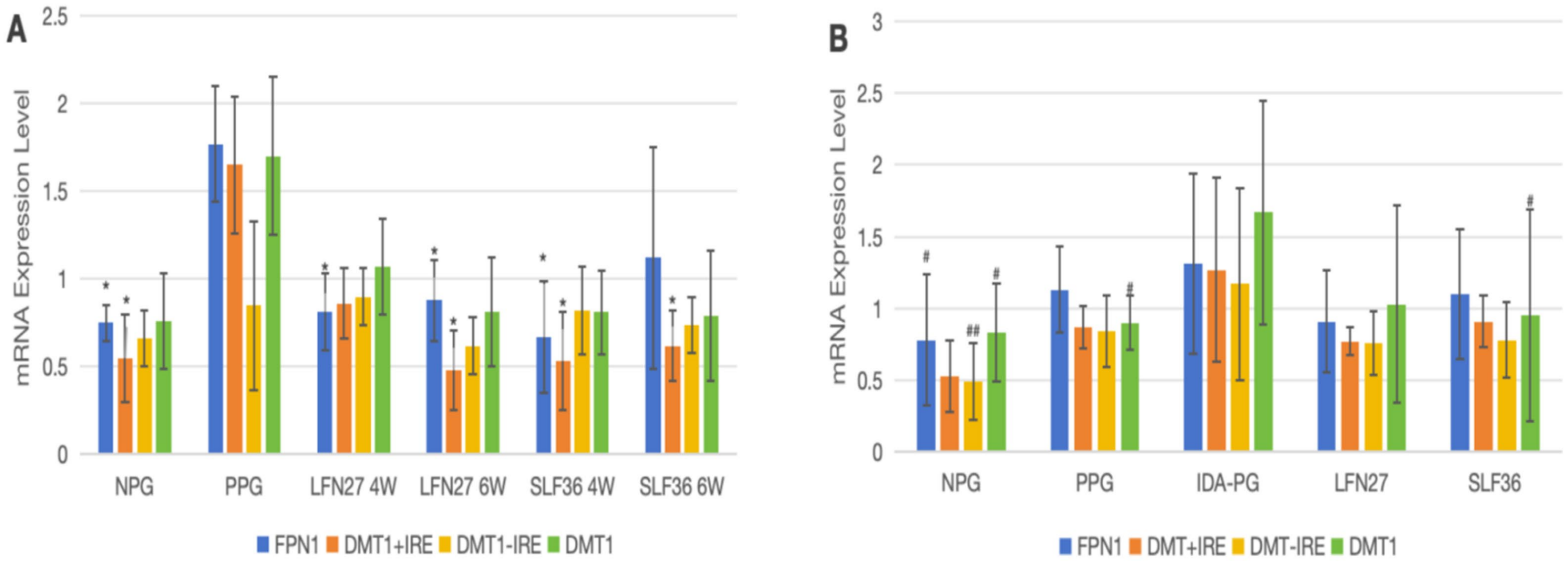
Effects of iron supplements on mRNA expression of iron metabolism-related indicators in the small intestine of pregnant anemia rats. **(A)** Physiological pregnancy anemia model; **(B)** pregnancy anemia model after IDA. Compared with the physiological pregnancy group, *P < 0.05; **P < 0.01; compared with the IDA pregnancy group, ^#^*P* < 0.05; ^##^*P* < 0.01.

As presented in Figure 13, for mRNA expression of small intestine iron metabolism-related indicators, compared with the non-pregnant group, the physiological pregnancy group had significantly higher levels of FPN1 and DMT1 + IRE mRNA.

When compared to the physiological pregnancy group, the four-week and six-week pre-administration LFN and SLF groups showed a significant decrease in small intestine FPN1 mRNA levels (*p* < 0.05). The six-week pre-administration LFN group exhibited a significant decrease in small intestine DMT1 + IRE mRNA levels (*p* < 0.05), as did the four-week and six-week pre-administration SLF groups (*p* < 0.05).

Feeding a low-iron diet to induce IDA in pregnant rats further aggravated the changes in all indicators. Compared to the non-pregnant group, the expression of FPN1, DMT, and DMT1 + IRE mRNA was significantly elevated (*p* < 0.05 or *p* < 0.01).

When compared to the IDA-pregnant group, there were no differences in FPN1 mRNA, DMT + IRE mRNA, or DMT − IRE mRNA levels between the LFN and SLF groups. However, the SLF group showed a significant decrease in DMT1 mRNA levels (*p* < 0.05).

**Figure 14:**
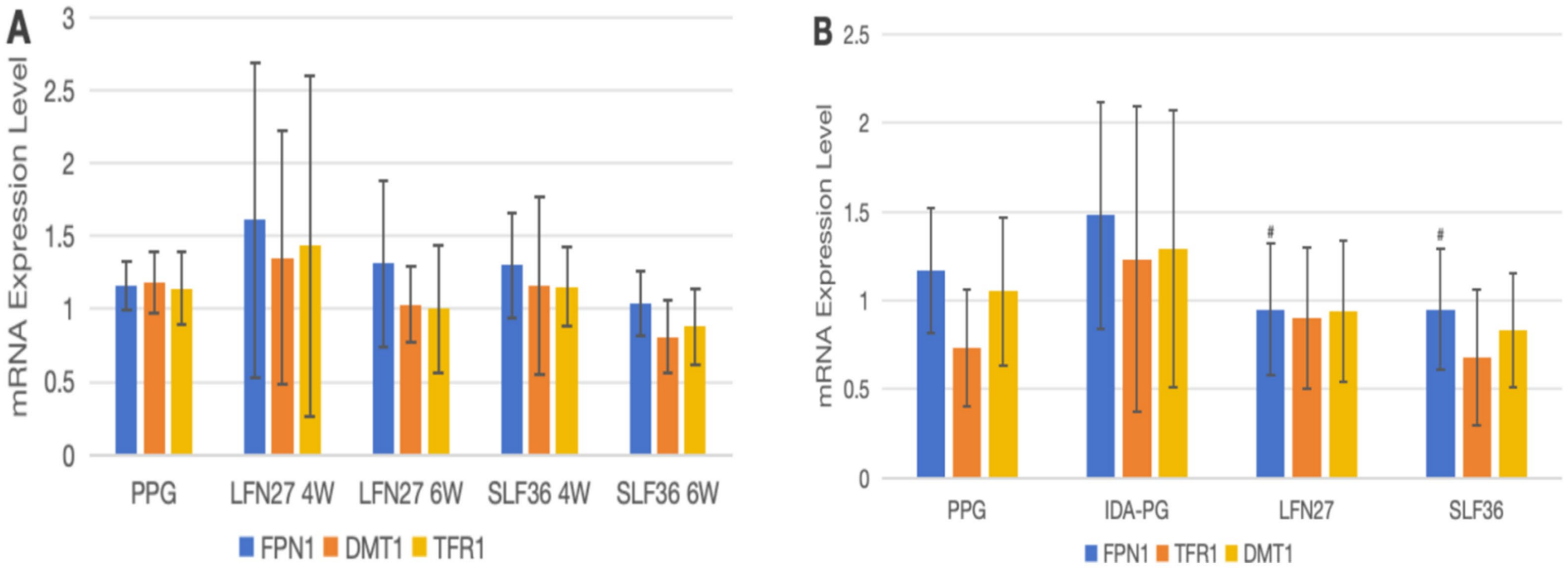
Effects of iron supplements on mRNA expression of iron metabolism-related indicators in the placenta of pregnant anemic rats. **(A)** Physiological pregnancy anemia model; **(B)** pregnancy anemia model after IDA. Compared with the IDA pregnancy group, ^#^*P* < 0.05.

As shown in Figure 14, for mRNA expression of placental iron metabolism-related indicators, when compared to the physiological pregnancy group, there were no statistically significant differences in the expression of FPN1, DMT1, and TFR1 mRNA in the LFN and SLF groups (*p* > 0.05).

There was a certain increasing trend in the gene expression levels of the IDA-pregnant group compared to the physiological pregnancy group, although the differences were not statistically significant (*p* > 0.05). When compared to the IDA-pregnant group, the LFN and SLF groups showed a significant decrease in placental FPN1 mRNA levels (*p* < 0.05).

## 4 Discussion

According to data from the WHO (10), IDA affects approximately 56 million pregnant women worldwide. As pregnancy progresses and the fetus undergoes rapid growth, the maternal demand for iron surges. However, daily dietary intake often fails to meet this increased demand, leading to the development of IDA (11). Simultaneously, the hemodilution state caused by an increase in maternal blood volume during pregnancy further exacerbates the risk of anemia (12). The consequences of IDA during pregnancy pose a more pronounced threat to both maternal and fetal health (13–16).

Gestational anemia is associated with an elevated risk of perinatal complications (17, 18). Pregnant women with anemia may face an increased likelihood of requiring blood transfusions, experiencing perinatal hemorrhage, preeclampsia, placental abruption, thyroid dysfunction, and even an elevated risk of postpartum heart failure and mortality (19, 20). For the fetus or newborn, inadequate iron stores can result in pre-term birth, low birth weight, and poor postnatal health outcomes. Moreover, it may have adverse effects on cognitive, motor, and emotional development (21, 22).

Although the detrimental effects of iron deficiency during pregnancy are widely recognized, and the demand for iron progressively increases as pregnancy advances (14, 23), the optimal approach to iron supplementation remains a subject of ongoing research and debate.

In this study, a physiological pregnancy model was established by mating female and male SD rats. Given that some women may already suffer from IDA prior to pregnancy due to factors such as dietary imbalance and unhealthy lifestyle habits (14, 23, 24), and their condition tends to worsen during pregnancy, thereby increasing pregnancy-related risks, based on existing pharmacological experimental foundations (4, 25, 26), we adopted a method of feeding rats with a simple iron-deficient diet to induce IDA before mating, thus establishing a pregnant rat model under IDA conditions.

This discovery is consistent with the clinical reports (27, 28) on abnormal blood routine and decreased serum iron metabolism indicators in pregnant women with anemia. This is also consistent with the research results of Gao et al. (29). After establishing a rat IDA model and mating to GD20, the blood routine of the IDA induced pregnant rat model further decreased compared to physiological pregnant rats, and the degree of tissue and serum iron deficiency intensified, indicating the successful establishment of the IDA induced pregnancy anemia model. This is consistent with the results of clinical studies (30) and pharmacological experiments (31). The results of this study suggest that pregnancy can lead to a decrease in stored iron and serum iron levels in the body, thereby causing anemia. Moreover, pregnancy following IDA further aggravates the iron-deficient status in both the serum and tissues.

To explore the effectiveness of preventive iron supplementation strategies, two commonly used oral iron supplements, SLF and LFN, were selected for research in clinical practice. The preliminary research of the research group found that pre-administration for 2 weeks did not significantly improve the iron deficiency status of rats. Based on this, the optimized administration plans adopted intervention strategies of pre-administration for 4 and 6 weeks.

The results indicated that, compared to the physiological pregnancy group, rats in the six-week pre-administered LFN and SLF groups showed an increase in RBC, HGB, and HCT, but the differences were not statistically significant, which is similar to the results of clinical studies (12). Meanwhile, in the SLF groups with four-week and six-week pre-administration, SI, FE, TSAT, and tissue iron content significantly increased. This suggests that preventive oral iron supplementation may be a suitable measure for reasonable pre-pregnancy iron supplementation in clinical practice (12) and can improve the iron status in a physiological gestational anemia model.

Further research revealed that oral iron supplements could significantly improve blood routine parameters and serum iron metabolism indicators in pregnant rats with IDA, while also increasing tissue iron content. There was no significant decrease in the number of pregnant rats in the pre-administration group, but both the SLF and LFN groups showed a decrease in implantation numbers after 6 weeks of pre-administration. The SLF group also had a decrease in corpus luteum number, live births, uterine fetal weight, and a slight increase in implantation mortality rate. The mean number of placental heavy nests in the LFN group decreased. This discovery is consistent with clinical reports on the adverse effects of excessive iron supplementation on fetal growth (32), but the specific mechanism still needs to be further explored. In the post-IDA pregnancy model, the number of pregnancies in the SLF group rats pretreated for 6 weeks decreased, indicating that long-term iron supplementation affects fertility, and further research is needed to confirm or verify this.

In the pregnancy model following IDA, a decrease in the number of pregnant rats was noted in the six-week pre-administered SLF group. This may be due to the limitations of iron supplementation or the constraints of the sample size, and further research is needed to confirm or validate this observation.

When LFN and SLF were administered to pregnant rats following IDA, it was found that there were no significant differences in the number of corpora lutea, implantation sites, implantation mortality, live fetuses, and uterine fetal weight between the two supplement-administered groups and the physiological pregnancy group. However, in the LFN group, the average weight per litter and the average crown–rump length per litter of the female offspring showed a significant increase. This indicates that iron supplements not only effectively improve pregnancy-related anemia following IDA but may also promote the weight and crown–rump length development of fetuses in pregnancies following IDA. This finding further emphasizes the importance of timely and appropriate iron supplementation for iron-deficient pregnant women in maintaining maternal iron homeostasis, ensuring the effective utilization of iron by the embryo, and promoting healthy fetal development, which is in line with the viewpoints in existing literature (33–35).

In view of this, it is recommended that close attention be paid to the iron status of women during pregnancy, and relevant anemia indicators be regularly examined. Once insufficient iron reserves or anemia is diagnosed, immediate measures should be taken, such as optimizing the dietary structure or supplementing with oral iron supplements, to ensure that the maternal serum iron level meets the needs of fetal growth and development, thereby reducing the risks of adverse pregnancy outcomes and fetal developmental abnormalities.

It is generally believed that iron overload leads to high oxidative stress (36, 37), but iron deficiency anemia also exhibits high levels of oxidative stress (38). This discovery found that pregnant rats with IDA have a high level of oxidative stress in their bodies, suggesting that iron deficiency may cause oxidative stress in the body (39, 40). Epidemiological and clinical research evidence also suggests a correlation between levels of oxidants and anemia (41).

The results of this study showed that in the livers of physiological pregnant rats, the levels of oxygen-free radical scavengers SOD and GPX1 significantly decreased, while the levels of the oxidative product MDA increased, and the T-AOC also weakened. In pregnant rats following IDA, the changes in oxidative stress indicators were further exacerbated. After intervention with iron-supplementation drugs, the levels of T-AOC, SOD, GPX1, and MDA in the livers of animals in the LFN and SLF groups showed varying degrees of reversal. This suggests that both physiological gestational anemia and pregnancy following IDA can stimulate the production of oxidative stress responses in the body, and pregnancy following IDA can aggravate the oxidative stress state within the body. After intervention with iron supplements, the antioxidant capacity of the livers shows varying degrees of recovery.

Iron is absorbed into intestinal cells through DMT1 (42), stored in the form of Fe^2+^, or oxidized by FPN1 and combined with transferrin to enter the bloodstream (43, 44). Liver ferritin (HAMP) binds and degrades FPN1, inhibiting iron release (45–47). Downregulation of HAMP during pregnancy enhances intestinal iron absorption and DMT1 transport (48).

The results of this study indicated that compared with non-pregnant rats, the expression of hepatic HAMP mRNA in physiological pregnant rats significantly decreased, while the expressions of small intestinal FPN1 and DMT1 + IRE mRNA significantly increased, and the serum level of TF also showed a significant increase. In pregnant rats following IDA, the changes in the expressions of hepatic HAMP, FPN1, and TFR1 mRNA, as well as small intestinal FPN1 and DMT1 mRNA, and the levels of serum TF and TFR1 were further exacerbated, which is consistent with the results reported by Cornock et al. (49). After intervention with iron supplements, the changes in the expressions of hepatic HAMP and small intestinal FPN1 and DMT1 + IRE mRNA, as well as the serum level of TF in physiological pregnant rats, could be significantly reversed. However, the effects on the expressions of the above – mentioned genes in anemic rats following IDA were weakened, and there was no statistical difference compared with physiological pregnant rats. This suggests that the mechanism by which iron supplements improve pregnancy-related anemia may be related to their ability to reduce the expression of HAMP mRNA in animals with the pregnancy model following IDA, enhance intestinal iron absorption and transport, so as to meet the iron metabolism balance of the fetus and adapt to the changes in maternal iron metabolism.

In addition, serum EPO is an endogenous glycoprotein hormone in humans that stimulates erythropoiesis (50). In this study, it was found that interventions with l LFN and SLF could reduce the EPO levels in both physiological pregnant rats and pregnant rats following IDA. However, in the pregnancy model following IDA, the effect of these interventions on the elevated expression of HAMP mRNA was not significant. This further supports the hypothesis that iron supplements can influence systemic iron metabolism and improve the anemia state by regulating HAMP and EPO.

This experiment suggests that long-term pre-iron supplementation may reduce fertility or be associated with iron overload and iron death. The impact of iron overload/iron death on infertility has been widely studied (51, 52). Iron-induced cell death is a special type of cell death triggered by iron accumulation and lipid peroxidation (53), ultimately leading to an increase in ROS and cell death (54). In women, it may impair fertility by interfering with gonadal function, damaging the ovaries, and weakening endometrial receptivity (52). However, oral iron supplements are currently a routine treatment for gestational IDA, and clinical large-scale studies have found that they do not significantly affect the conception and embryonic development of rats (55). Therefore, we still recommend timely and moderate supplementation of oral iron supplements during pregnancy when iron deficiency and IDA occur.

Iron, as a core component of key biological molecules, is indispensable for maintaining the morphology of red blood cells, oxygen transport, and immune function (23). Although the specific mechanisms between iron status and human reproduction have not been fully elucidated in current research, it has been shown that both iron deficiency and iron overload can have adverse effects on health. Thus, for women preparing for pregnancy, if they do not have anemia and have normal iron stores, blind or excessive iron supplementation should be avoided. During pregnancy, serum iron levels should be regularly monitored, and iron-supplementation strategies should be flexibly adjusted according to the actual situation to ensure that the iron requirements of both the mother and the fetus are met while effectively avoiding the risks associated with abnormal serum iron levels.

Although this study provides valuable insights into the effectiveness and potential mechanisms of different iron supplementation strategies by establishing IDA rat models of physiological anemia during pregnancy and post-IDA pregnant rats, there are still some limitations that need to be clarified: First, the rat models used have significant differences from humans in terms of pregnancy cycle, iron metabolism rate, and placental structure. Additionally, the IDA model induced solely by iron deficiency diet mainly simulates nutritional iron deficiency and fails to cover complex clinical causes such as absorption disorders or chronic blood loss, limiting the extrapolation of results. Second, potential negative effects of pre-iron supplementation on some reproductive parameters have been observed, but due to sample size limitations, the statistical power is insufficient and requires larger sample studies for confirmation. Finally, the depth of mechanism exploration is limited: There is a lack of direct evidence linking the speculated iron-supplementation-related decline in fertility with iron overload/iron death. Meanwhile, the inability to directly evaluate placental iron transporter activity and fetal iron status hinders a deeper understanding of how iron supplementation specifically affects maternal fetal iron allocation and fetal development. In response to the above limitations, subsequent research will focus on in-depth mechanism analysis and clinical translation. Firstly, iron death inhibitor intervention experiments will be conducted to verify the direct causal relationship between iron-induced fertility decline and iron; secondly, using isotope tracing and protein-activity-detection techniques, the function of placental iron transporters and fetal iron status are dynamically evaluated to elucidate the specific effects of iron supplementation on maternal fetal iron distribution. We will further combine multi-omics analyses to screen key pathways regulating iron homeostasis and draw a correlation network between iron threshold and fetal developmental abnormalities. Ultimately, based on the above mechanism, an individualized iron supplementation program guided by iron metabolism markers will be developed as a reproductive protection strategy.

## Data Availability

The original contributions presented in the study are included in the article/Supplementary material, further inquiries can be directed to the corresponding authors.
