# Correction: Differential effects of prophylactic iron supplementation on physiological gestational anemia and post IDA gestational anemia: a study based on a rat model

**DOI:** 10.3389/fnut.2025.1704849

**Published:** 2025-11-17

**Authors:** Zelin Zhang, Limin Lai, Ziping Liu, Sili Liu, Liping Qu, Wenjun Zou

**Affiliations:** Chengdu University of Chinese Medicine, Chengdu, China

**Keywords:** pregnancy anemia, iron deficiency anemia, iron metabolism, iron supplements, fertility

There was a mistake in Figure 1 as published. The Figure 1 part label images C & D were missing. The corrected figure and its caption appear below.

**Figure 1:**
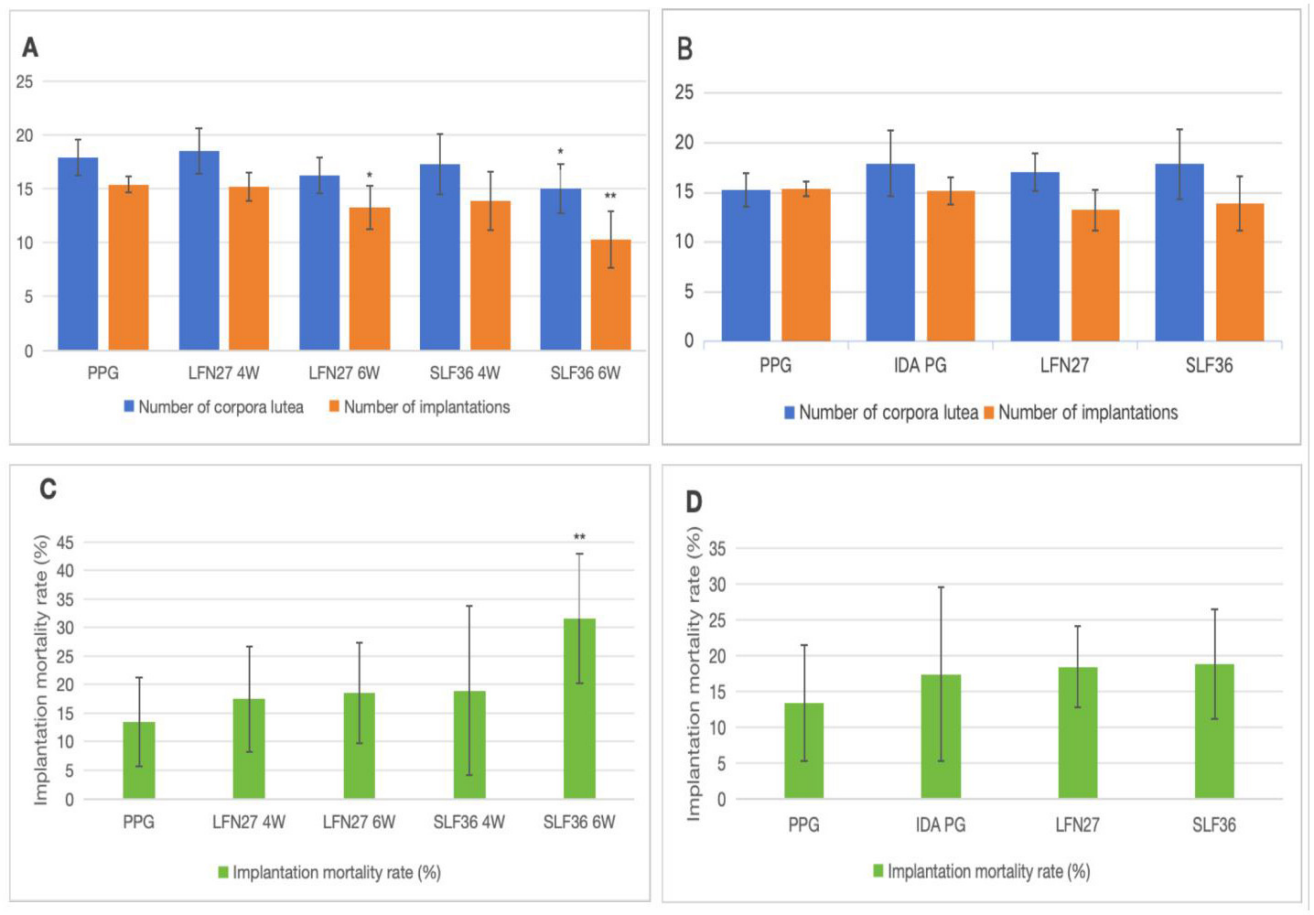
Effects of iron supplements on the fertility of pregnant anemia rats. **(A, C)** Physiological pregnancy anemia model. **(B, D)** Pregnancy anemia model after IDA. Compared with the physiological pregnancy group, *P < 0.05; **P < 0.01.

There was a mistake in Figure 2 as published. The Figure 2 part label images C and D were missing. The corrected figure and its caption appear below.

**Figure 2:**
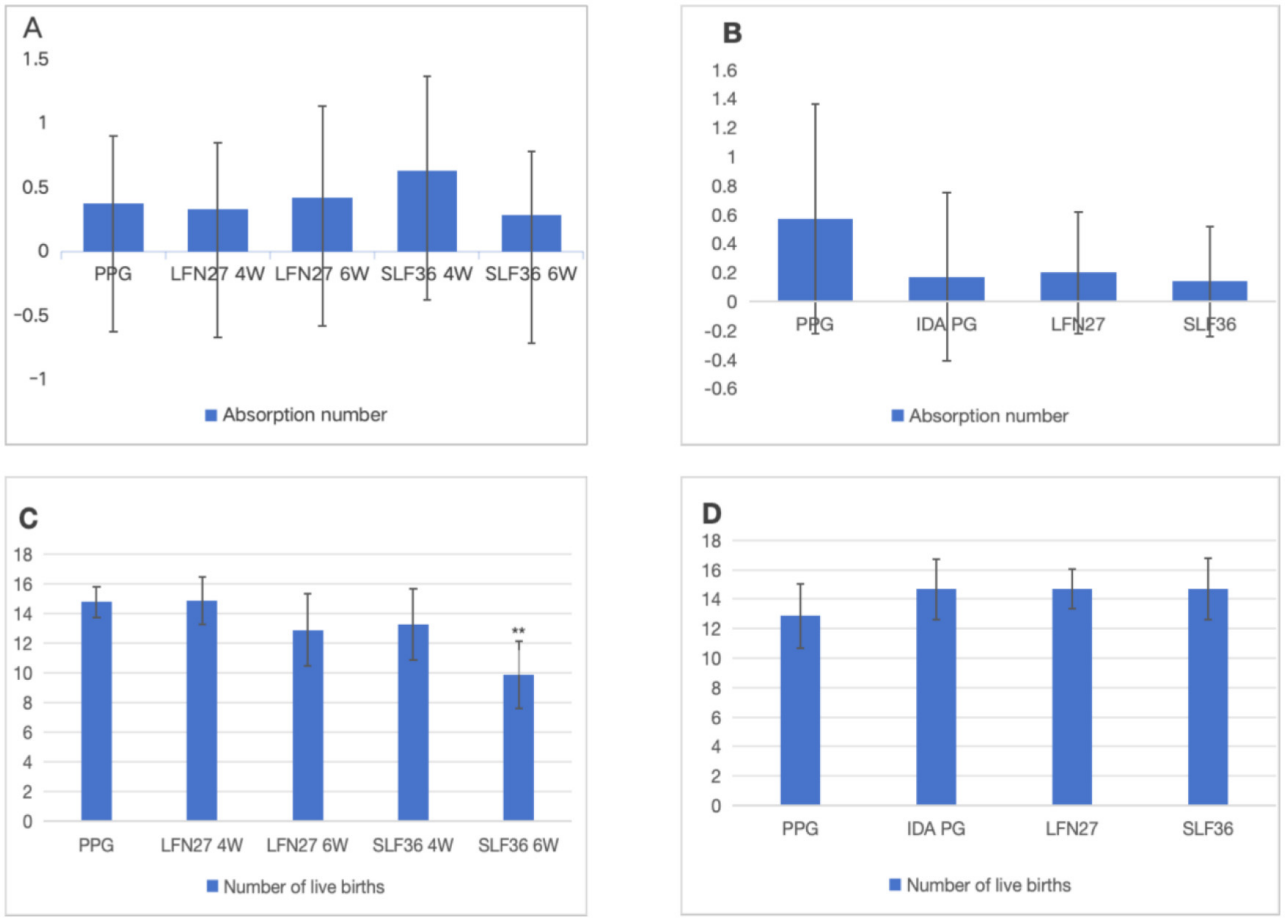
Effect of iron supplements on the number of embryos in pregnant anemic rats. **(A, C)** Physiological pregnancy anemia model. **(B, D)** Pregnancy anemia model after IDA. Compared with the physiological pregnancy group, **P < 0.01.

There was a mistake in Figure 3 as published. In Figures 3C, D images, the symbol “#” should have been used in the pregnancy anemia model after IDA. The corrected Figure 3 appears below.

**Figure 3:**
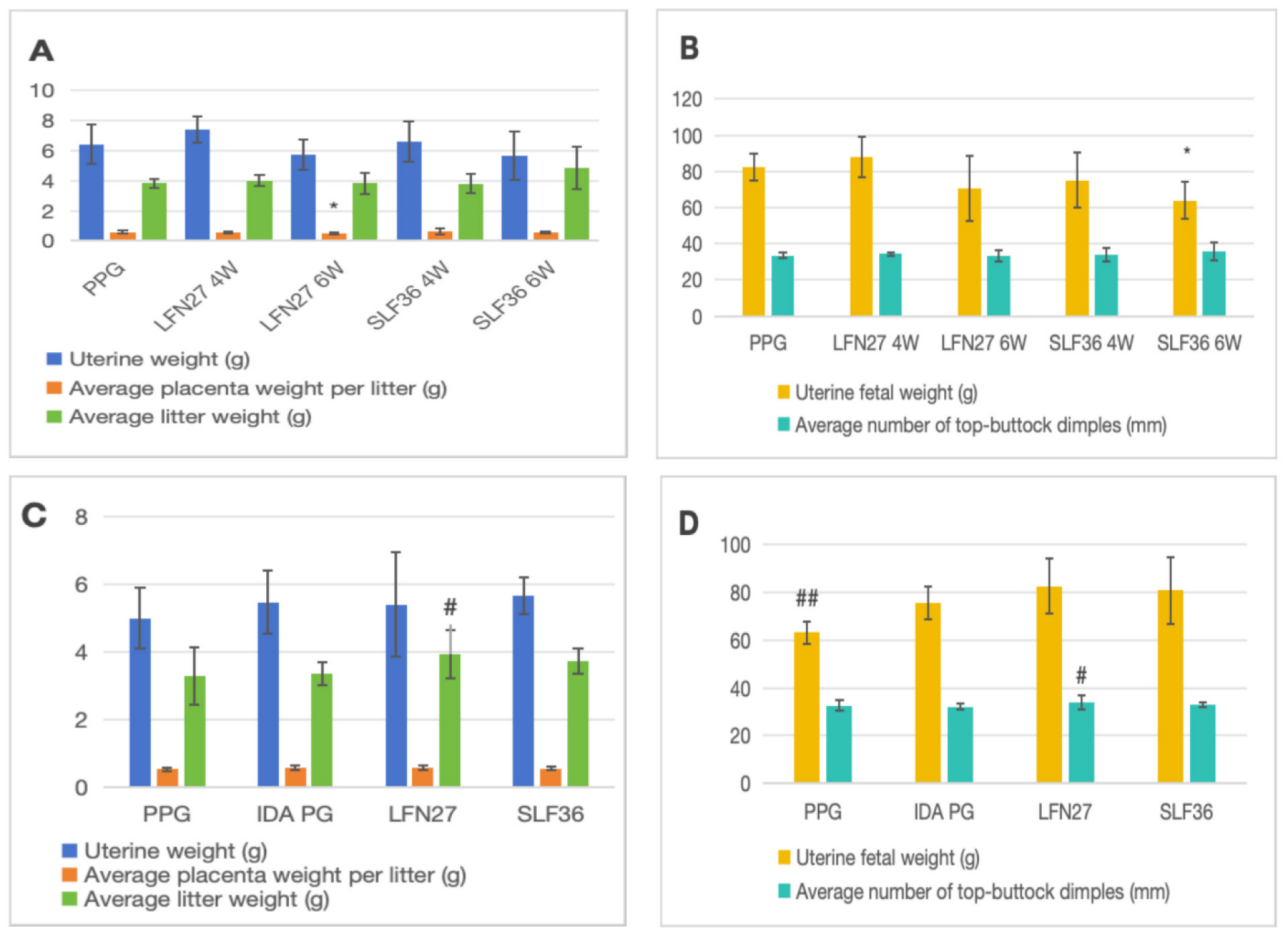
Effects of iron supplements on embryo and offspring development in pregnant anemia rats. **(A, B)** Physiological pregnancy anemia model. **(C, D)** Pregnancy anemia model after IDA. Compared with the physiological pregnancy group, *P < 0.05; compared with the IDA pregnancy group, ^#^*P* < 0.05; ^*##*^*P* < 0.01.

There was a mistake in Figure 6 as published. In Figures 6C, D images, the symbol “#” should have been used in the pregnancy anemia model after IDA. The corrected Figure 6 appears below.

**Figure 6:**
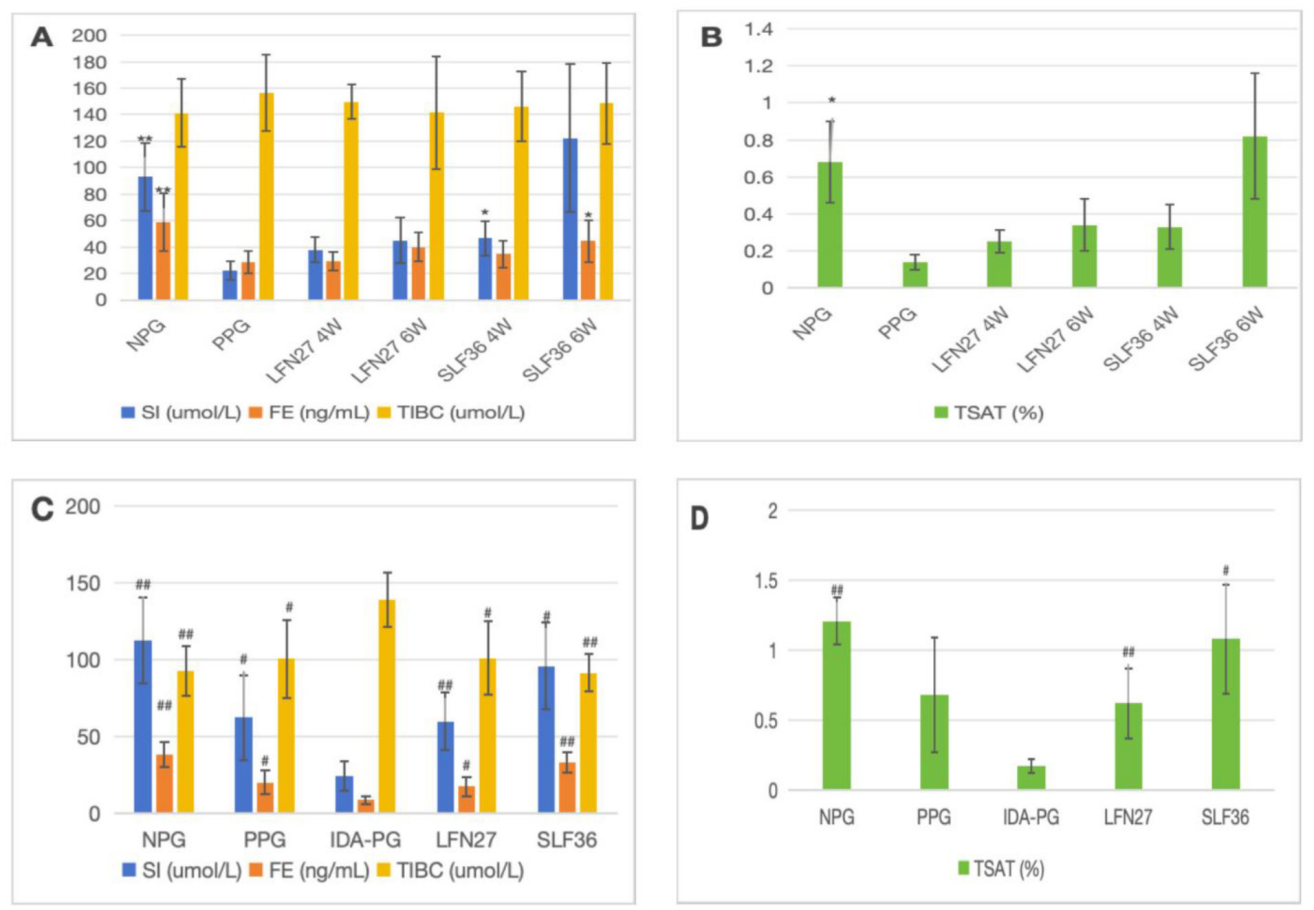
Effects of GD20 iron supplement on serum iron-related indicators in pregnant anemia rats. **(A, B)** Physiological pregnancy anemia model. **(C, D)** Pregnancy anemia model after IDA. Compared with the physiological pregnancy group, *P < 0.05; **P < 0.01. Compared with the IDA pregnancy group, ^#^*P* < 0.05; ^*##*^*P* < 0.01.

There was a mistake in Figure 7 as published. In Figure 7B image, the symbol “#” should have been used in the pregnancy anemia model after IDA. The corrected Figure 7 appears below.

**Figure 7:**
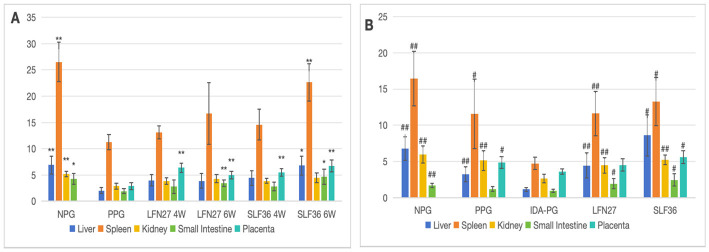
Effect of GD20 iron supplement on tissue iron content in pregnant anemic rats. **(A)** Physiological pregnancy anemia model. **(B)** Pregnancy anemia model after IDA. Compared with the physiological pregnancy group, *P < 0.05; **P < 0.01. Compared with the IDA pregnancy group, ^#^*P* < 0.05; ^##^*P* < 0.01.

There was a mistake in Figure 8 as published. In Figure 8D, CPXI on the vertical axis should be GPX1. The corrected Figure 8 appears below.

**Figure 8:**
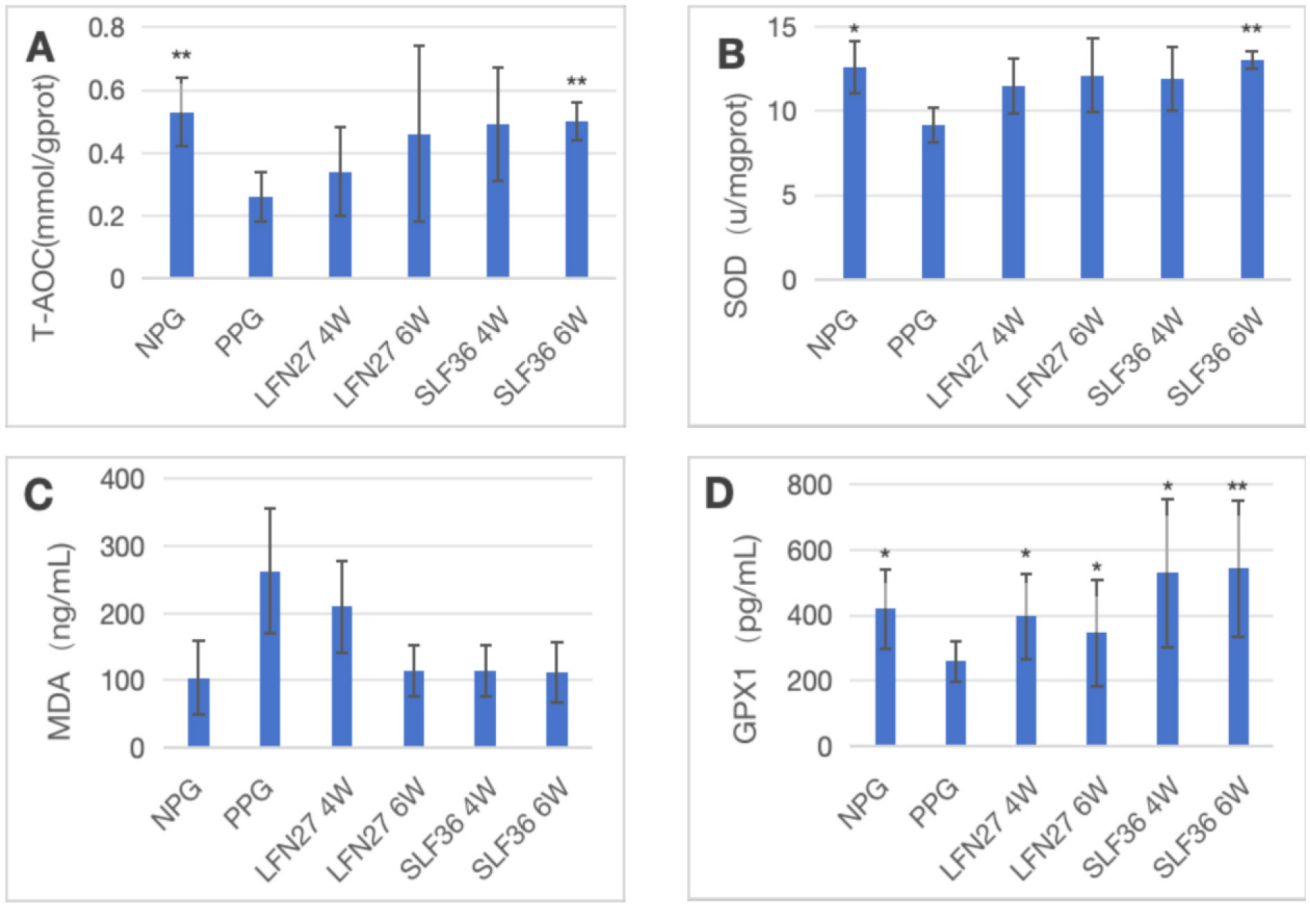
Effects of GD20 iron supplement on liver oxidative stress related indicators in physiological pregnancy group. **(A)** T-AOC; **(B)** SOD; **(C)** MDA; **(D)** GPX1. Compared with the physiological pregnancy group, *P < 0.05; **P < 0.01.

There was a mistake in Figure 9 as published. In Figure 9D, CPXI on the vertical axis should be GPX1. The corrected Figure 9 appears below.

**Figure 9:**
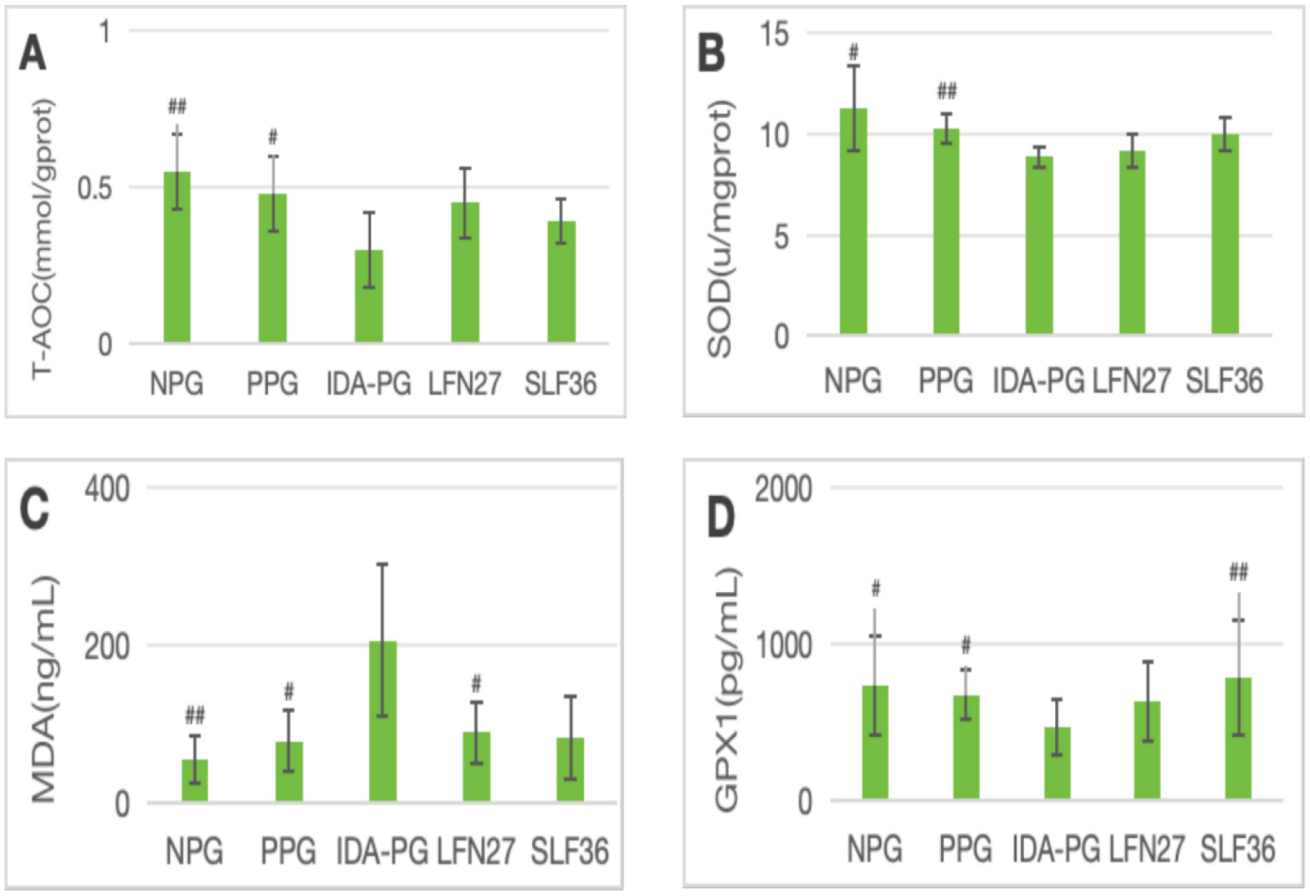
Effects of GD20 iron supplement on liver oxidative stress related indicators in pregnancy anemia model after IDA. **(A)** T-AOC; **(B)** SOD; **(C)** MDA; **(D)** GPX1. Compared with the IDA pregnancy group, ^#^*P* < 0.05; ^##^*P* < 0.01.

There was a mistake in Figure 11 as published. In Figure 11B image, the symbol “#” should have been used in the pregnancy anemia model after IDA. The corrected Figure 11 appears below.

**Figure 11:**
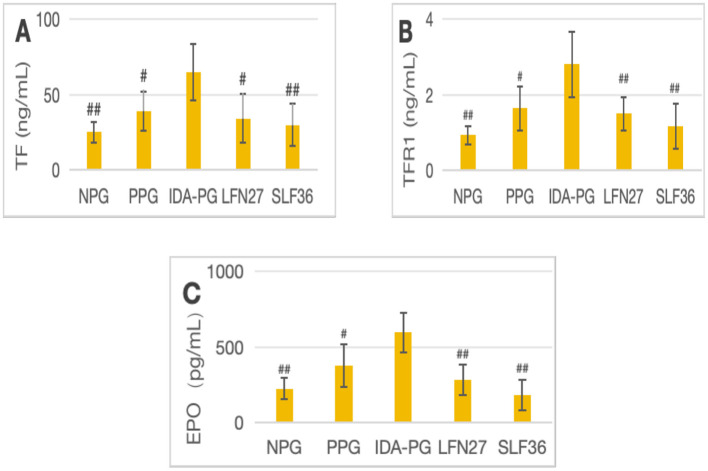
Effects of LFN and SLF on serum iron metabolism-related indicators in pregnancy anemia model after IDA. **(A)** TF; **(B)** TFR1; **(C)** EPO. Compared with the IDA pregnancy group, ^#^*P* < 0.05; ^##^*P* < 0.01.

There was a mistake in Figure 12 as published. In Figure 12B image, the symbol “#” should have been used in the pregnancy anemia model after IDA. The corrected Figure 12 appears below.

**Figure 12:**
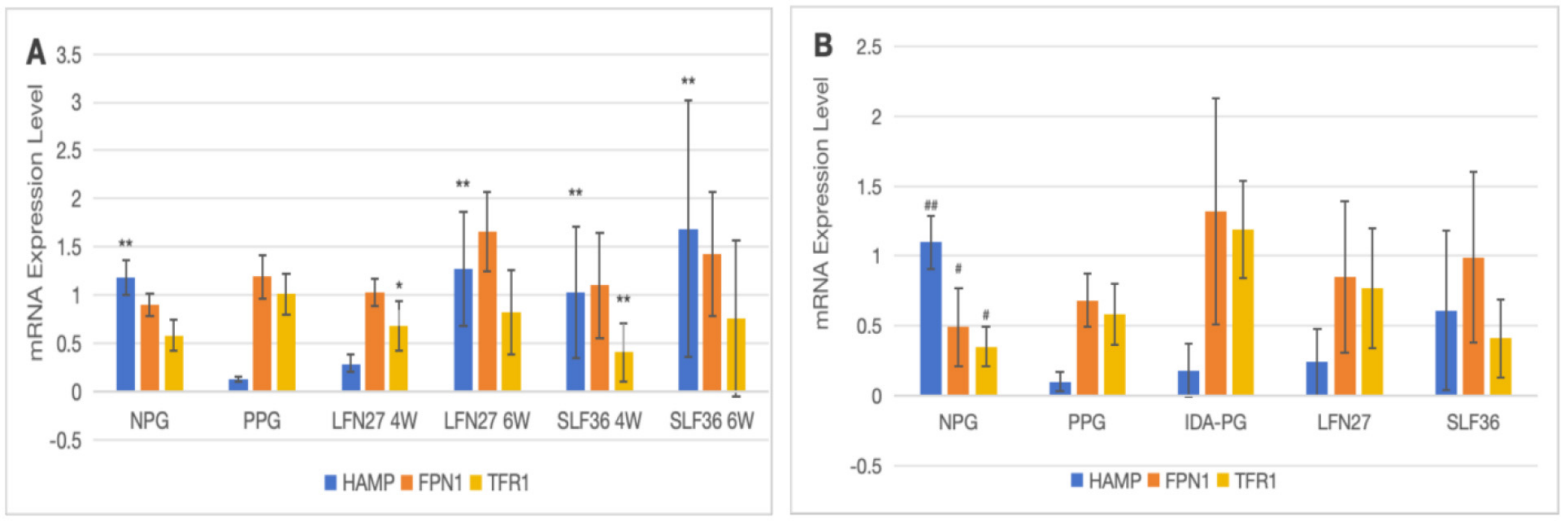
Effects of iron supplements on mRNA expression of liver iron metabolism-related indicators in pregnant anemic rats. **(A)** Physiological pregnancy anemia model; **(B)** pregnancy anemia model after IDA. Compared with the physiological pregnancy group, *P < 0.05; **P < 0.01; compared with the IDA pregnancy group, ^#^*P* < 0.05; ^##^*P* < 0.01.

There was a mistake in Figure 13 as published. In Figure 13B image, the symbol “#” should have been used in the pregnancy anemia model after IDA. The corrected Figure 13 appears below.

**Figure 13:**
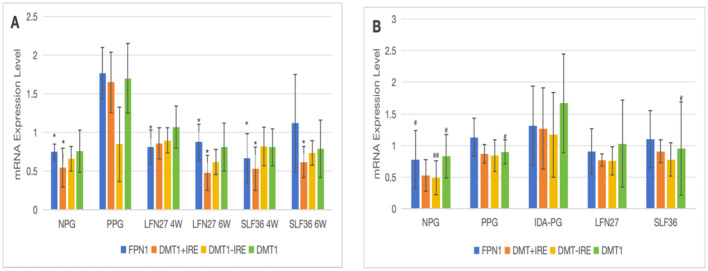
Effects of iron supplements on mRNA expression of iron metabolism-related indicators in the small intestine of pregnant anemia rats. **(A)** Physiological pregnancy anemia model; **(B)** pregnancy anemia model after IDA. Compared with the physiological pregnancy group, *P < 0.05; **P < 0.01; compared with the IDA pregnancy group, ^#^*P* < 0.05; ^##^*P* < 0.01.

There was a mistake in Figure 14 as published. In Figure 14B image, the symbol “#” should have been used in the pregnancy anemia model after IDA. The corrected Figure 14 appears below.

**Figure 14:**
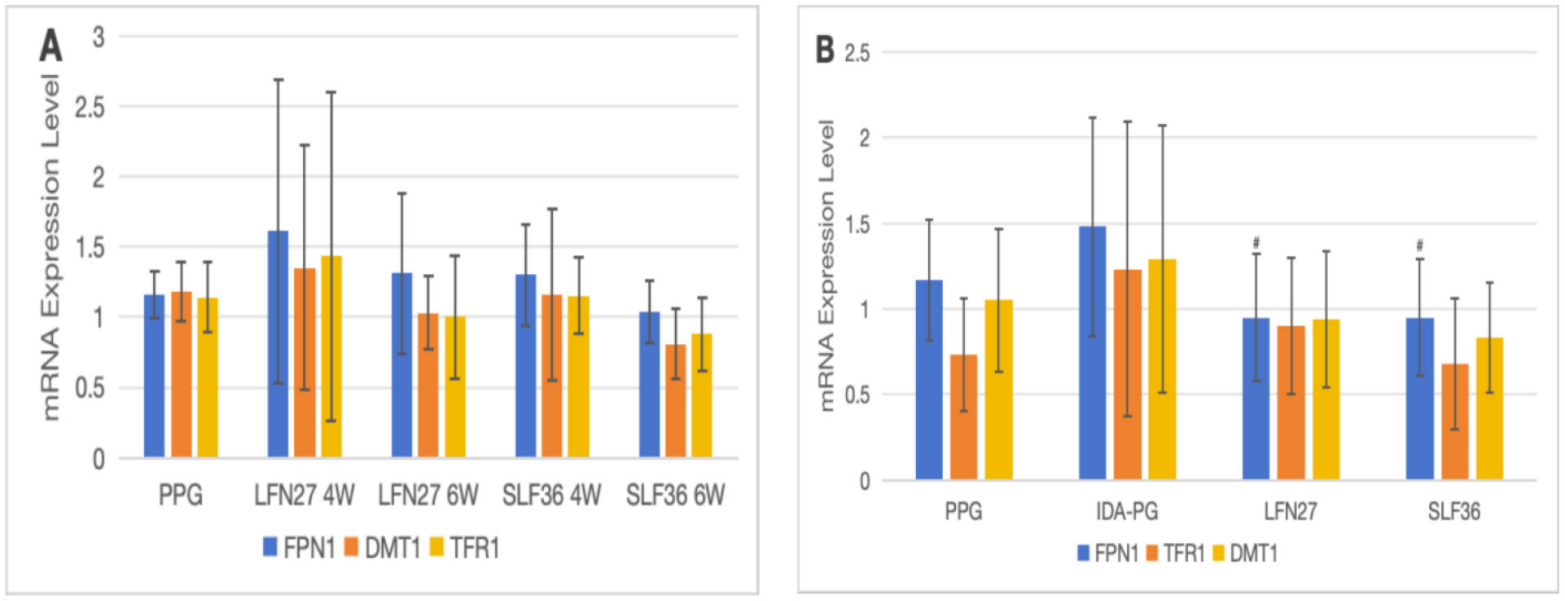
Effects of iron supplements on mRNA expression of iron metabolism-related indicators in the placenta of pregnant anemic rats. **(A)** Physiological pregnancy anemia model; **(B)** pregnancy anemia model after IDA. Compared with the IDA pregnancy group, ^#^*P* < 0.05.

Supplementary Table 1 was erroneously published with the original version of this paper. The file has now been replaced.

The original version of this article has been updated.

