# Supplementary material for: Differential effects of prophylactic iron supplementation on physiological gestational anemia and post-IDA gestational anemia: a study based on a rat model
Source: Front Nutr. 2025 Sep 12;12:1650536. doi: 10.3389/fnut.2025.1650536 (PMC12465626; doi:10.3389/fnut.2025.1650536)
Supplement: Supplementary file 1 [file Table_1.DOCX]

Supplement Table

Table 1 Effects of Iron Supplements on the Fertility of Pregnant Anemia Rats

| Model | Group | N | Number of pregnancies | Pre administration (week) | Luteal number | Number of implantation sites | Implantation mortality rate (%) |
| --- | --- | --- | --- | --- | --- | --- | --- |
| Physiological pregnancy anemia model | Physiological pregnancy group | 10 | 8 | 0 | 17.88±1.64 | 15.38±0.74 | 13.46±7.74 |
|  | LFN group | 10 | 6 | 4 | 18.50±2.07 | 15.17±1.33 | 17.46±9.28 |
|  |  | 10 | 8 | 6 | 16.25±1.67 | 13.25±2.05^*^ | 18.51±8.82 |
|  | SLF group | 10 | 8 | 4 | 17.29±2.81 | 13.86±2.73 | 18.93±14.77 |
|  |  | 10 | 7 | 6 | 15.00±2.31^*^ | 10.29±2.63^**^ | 31.62±11.34^**^ |
| Pregnancy anemia model after IDA | Physiological pregnancy group | 14 | 7 | 0 | 15.29±1.70 | 13.57±1.62 | 10.96±8.11 |
|  | IDA pregnancy group | 14 | 12 | 0 | 17.92±3.29 | 15.17±2.04 | 14.10±12.09 |
|  | LFN group | 14 | 10 | 7 | 17.00±1.89 | 14.90±1.37 | 12.05±5.64 |
|  | SLF group | 14 | 7^#^ | 7 | 17.86±3.53 | 15.00±2.00 | 14.98±7.64 |

Table 2 Effect of iron supplements on the number of embryos in pregnant anemic rats

| Model | Group | N | Pre administration (week) | Number of absorbed fetuses | Early stillbirth count | Number of late stage stillbirths | Number of live births |
| --- | --- | --- | --- | --- | --- | --- | --- |
| Physiological pregnancy anemia model | Physiological pregnancy group | 8 | 0 | 0.38±0.52 | 0.13±0.35 | 0.13±0.35 | 14.75±1.04 |
|  | LFN group | 6 | 4 | 0.33±0.52 | 0.00±0.00 | 0.00±0.00 | 14.83±1.60 |
|  |  | 8 | 6 | 0.42±0.72 | 0.00±0.00 | 0.00±0.00 | 12.88±2.42 |
|  | SLF group | 8 | 4 | 0.63±0.74 | 0.00±0.00 | 0.00±0.00 | 13.25±2.38 |
|  |  | 7 | 6 | 0.29±0.49 | 0.00±0.00 | 0.14±0.38 | 9.86±2.27^**^ |
| Pregnancy anemia model after IDA | Physiological pregnancy group | 7 | 0 | 0.57±0.79 | 0.00±0.00 | 0.14±0.38 | 12.86±2.19 |
|  | IDA pregnancy group | 12 | 0 | 0.17±0.58 | 0.00±0.00 | 0.33±0.49 | 14.67±2.06 |
|  | LFN group | 10 | 7 | 0.20±0.42 | 0.00±0.00 | 0.00±0.00 | 14.70±1.34 |
|  | SLF group | 7 | 7 | 0.14±0.38 | 0.00±0.00 | 0.14±0.38 | 14.71±2.06 |

Table 3: Effects of Iron Supplements on Embryo and Offspring Development in Pregnant Anemia Rats

| Model | Group | N | Pre administration (week) | Uterine weight (g) | Mean number of placenta accreta (g) | Fetal weight average per litter (g) | Mean number of long dimples on the top and buttocks (mm) |
| --- | --- | --- | --- | --- | --- | --- | --- |
| Physiological pregnancy anemia model | Physiological pregnancy group | 8 | 0 | 6.43±1.30 | 0.59±0.08 | 3.83±0.29 | 33.57±1.50 |
|  | LFN group | 6 | 4 | 7.42±0.85 | 0.56±0.08 | 4.01±0.36 | 34.34±1.12 |
|  |  | 8 | 6 | 5.75±1.00 | 0.49±0.05^*^ | 3.84±0.70 | 33.37±3.26 |
|  | SLF group | 8 | 4 | 6.59±1.33 | 0.60±0.20 | 3.81±0.66 | 33.74±3.83 |
|  |  | 7 | 6 | 5.66±1.59 | 0.58±0.06 | 4.84±1.42 | 35.78±4.68 |
| Pregnancy anemia model after IDA | Physiological pregnancy group | 7 | 0 | 4.99±0.89 | 0.52±0.05 | 3.29±0.85 | 32.60±2.26 |
|  | IDA pregnancy group | 12 | 0 | 5.47±0.95 | 0.56±0.07 | 3.36±0.34 | 32.21±1.45 |
|  | LFN group | 11 | 7 | 5.40±1.55 | 0.56±0.08 | **3.92±0.71^#^** | **33.98±2.78^#^** |
|  | SLF group | 7 | 7 | 5.67±0.54 | 0.54±0.05 | 3.72±0.36 | 33.12±1.06 |

、

Table 4 Blood routine of physiological pregnancy anemia rats treated with iron supplements

| Model | Group | Pre administration (week) | RBC | HGB | HCT |
| --- | --- | --- | --- | --- | --- |
| D42 | Unpregnant group^a^ | 0 | 7.03±0.88 | 136.25±15.63 | 41.34±5.62 |
|  | LFN group | 4 | 7.39±0.43 | 144.33±7.39 | 43.17±2.59 |
|  |  | 6 | 6.75±1.75 | 130.63±28.74 | 38.51±10.63 |
|  | SLF group | 4 | 6.60±1.27 | 128.50±16.87 | 38.68±5.67 |
|  |  | 6 | 6.96±0.37 | 135.57±6.32 | 41.03±2.01 |
| GD20 | Unpregnant group | 0 | 8.23±0.49^##^ | 152.50±6.76^#^ | 44.50±1.68^#^ |
|  | Physiological pregnancy group | 0 | 6.39±0.79 | 122.25±14.44 | 35.99±4.32 |
|  | LFN group | 4 | 5.73±0.59 | 116.33±4.50 | 34.35±1.79 |
|  |  | 6 | 7.18±1.36 | 135.63±24.94 | 39.93±7.27 |
|  | SLF group | 4 | 6.58±0.77 | 126.13±13.34 | 37.44±4.13 |
|  |  | 6 | 6.80±1.06 | 131.29±16.19 | 38.71±4.56 |

Table 5 Effects of Iron Supplements on Blood Routine of IDA Pregnant Rats

|  | Group | N | RBC（10^12^/L） | HGB（g/L） | HCT（%） |
| --- | --- | --- | --- | --- | --- |
| D49 | Unpregnant group^a^ | 24 | 7.53±0.47 | 141.25±7.86^**^ | 42.1±2.43^**^ |
|  | IDA pregnancy group | 14 | 7.48±0.32 | 129.07±6.41 | 39.08±1.69 |
|  | LFN group | 14 | 7.33±0.63 | 138.36±11.32 | 41.39±3.40 |
|  | SLF group | 14 | 7.50±0.35 | 141.29±7.39^**^ | 41.75±1.91^*^ |
| GD20 | Unpregnant group | 10 | 8.11±0.30^**^ | 146.8±5.88^**^ | 43.33±1.58^**^ |
|  | Physiological pregnancy group | 10 | 7.48±0.61^*^ | 140.8±11.84^**^ | 40.03±3.32^**^ |
|  | IDA pregnancy group | 12 | 6.47±0.43 | 109.91±6.67 | 31.74±2.33 |
|  | LFN group | 11 | 7.24±0.63 | 134.91±10.88^**^ | 39.08±3.41^**^ |
|  | SLF group | 7 | 7.29±0.65 | 136.86±8.57 ^**^ | 39.64±2.68^**^ |

Table 6 Effects of GD20 Iron Supplement on Serum Iron Related Indicators in Pregnant Anemia Rats

| Model | Group | Pre administration (week) | SI (umol/L) | FE (ng/mL) | TIBC (umol/L) | TSAT (%) |
| --- | --- | --- | --- | --- | --- | --- |
| Physiological pregnancy anemia model | Unpregnant group | 0 | 92.99±25.81^**^ | 58.57±21.77^**^ | 141.34±25.74 | 0.68±0.22^*^ |
|  | Physiological pregnancy group | 0 | 22.22±7.06 | 28.61±8.57 | 156.66±28.79 | 0.14±0.04 |
|  | LFN group | 4 | 37.88±9.56 | 29.34±7.18 | 149.78±12.77 | 0.25±0.06 |
|  |  | 6 | 44.79±17.14 | 40.03±11.04 | 141.64±42.60 | 0.34±0.14 |
|  | SLF group | 4 | 46.45±12.95^*^ | 34.68±10.34 | 146.02±26.39 | 0.33±0.12 |
|  |  | 6 | 122.35±56.14 | 44.34±15.50^*^ | 148.45±30.32 | 0.82±0.34 |
| Pregnancy anemia model after IDA | Unpregnant group | 0 | 112.75±27.87^##^ | 38.11±8.40^##^ | 92.72±15.99^##^ | 1.21±0.17^##^ |
|  | Physiological pregnancy group | 0 | 62.34±27.53^#^ | 20.03±7.94^#^ | 100.43±25.58^#^ | 0.68±0.41 |
|  | IDA pregnancy group | 0 | 23.99±9.64 | 8.47±2.54 | 138.74±17.72 | 0.17±0.05 |
|  | LFN group | 7 | 59.76±19.00^##^ | 17.35±6.27^#^ | 101.02±23.75^#^ | 0.62±0.25^##^ |
|  | SLF group | 7 | 95.79±28.24^#^ | 33.00±6.68^##^ | 91.34±12.04^##^ | 1.08±0.39^#^ |

Table 7 Effect of GD20 iron supplement on tissue iron content in pregnant anemic rats

| Model | Group | Pre administration (week) | Liver  （μmol/gprot） | Spleen（μmol/gprot） | Kidney  （μmol/gprot） | Small intestine  （μmol/gprot） | Placenta  （μmol/gprot） |
| --- | --- | --- | --- | --- | --- | --- | --- |
| Physiological pregnancy anemia model | Unpregnant group | 0 | 6.91±1.68^**^ | 26.52±3.77^**^ | 5.20±0.50^**^ | 4.26±1.02^*^ | — |
|  | Physiological pregnancy group | 0 | 2.05±0.58 | 11.27±1.44 | 2.91±0.57 | 1.91±0.46 | 2.88±0.62 |
|  | LFN group | 4 | 4.01±1.13 | 13.10±1.27 | 3.87±0.61 | 2.81±1.30 | 6.38±0.84^**^ |
|  |  | 6 | 3.87±1.39 | 16.68±5.86 | 4.31±0.74 | 3.41±0.60^**^ | 4.96±0.70^**^ |
|  | SLF group | 4 | 4.44±1.39 | 14.59±2.92 | 3.89±0.45 | 2.84±0.82 | 5.52±0.75^**^ |
|  |  | 6 | 6.79±1.84^*^ | 22.64±3.51^**^ | 4.46±0.89 | 4.69±1.43^*^ | 6.73±1.17^**^ |
| Pregnancy anemia model after IDA | Unpregnant group | 0 | 6.78±1.61^##^ | 16.45±3.79^##^ | 5.94±1.17^##^ | 1.65±0.31^##^ | —— |
|  | Physiological pregnancy group | 0 | 3.22±1.03^##^ | 11.57±4.79^#^ | 5.13±1.36^##^ | 1.18±0.35 | 4.87±0.82^#^ |
|  | IDA pregnancy group | 0 | 1.14±0.27 | 4.73±0.88 | 2.62±0.58 | 0.92±0.20 | 3.60±0.39 |
|  | LFN group | 7 | 4.44±1.72^##^ | 11.61±3.09^##^ | 4.45±1.07^##^ | 1.91±0.71^#^ | 4.50±0.83 |
|  | SLF group | 7 | 8.58±2.87^#^ | 13.28±3.31^#^ | 5.21±0.69^##^ | 2.44±0.86^#^ | 5.61±0.89^#^ |

Table 8 Effects of GD20 Iron Supplement on Liver Oxidative Stress Related Indicators in Pregnant Anemia Rats

| Model | Group | Pre administration (week) | T-AOC  (mmol/gprot) | SOD  (u/mgprot) | MDA  (ng/mL) | GPX1  (pg/mL) |
| --- | --- | --- | --- | --- | --- | --- |
| Physiological pregnancy anemia model | Unpregnant group | 0 | 0.53±0.11^**^ | 12.64±1.55^*^ | 103.43±54.56 | 419.35±122.38^*^ |
|  | Physiological pregnancy group | 0 | 0.26±0.08 | 9.19±1.04 | 262.97±93.09 | 259.76±60.86 |
|  | LFN group | 4 | 0.34±0.14 | 11.51±1.63 | 209.81±68.37 | 397.06±129.49^*^ |
|  |  | 6 | 0.46±0.28 | 12.14±2.18 | 180.50±63.92 | 346.77±162.26^*^ |
|  | SLF group | 4 | 0.49±0.18 | 11.93±1.89 | 113.92±37.25 | 528.91±227.12^*^ |
|  |  | 6 | 0.50±0.06^**^ | 13.08±0.52^**^ | 111.87±44.47 | 542.73±208.28^**^ |
| Pregnancy anemia model after IDA | Unpregnant group | 0 | 0.55±0.12^##^ | 11.27±2.08^#^ | 56.29±29.91^##^ | 738.49±314.22^#^ |
|  | Physiological pregnancy group | 0 | 0.48±0.12^#^ | 10.29±0.76^##^ | 78.96±38.49^#^ | 680.88±159.02^#^ |
|  | IDA pregnancy group | 0 | 0.30±0.12 | 8.88±0.51 | 206.72±96.50 | 470.55±177.69 |
|  | LFN group | 7 | 0.45±0.11 | 9.18±0.84 | 89.76±37.87^#^ | 635.57±254.57 |
|  | SLF group | 7 | 0.39±0.07 | 10.02±0.79 | 83.69±52.58 | 790.81±363.41^##^ |

Table 9 Effects of LFN and SLF on serum iron metabolism related indicators in pregnant anemia rats

| Model | Group | Pre administration (week) | TF (ng/mL) | TFR1(ng/mL) | EPO (pg/mL) |
| --- | --- | --- | --- | --- | --- |
| Physiological pregnancy anemia model | Unpregnant group | 0 | 51.33±14.39^*^ | 2.58±1.12 | 288.35±82.68^**^ |
|  | Physiological pregnancy group | 0 | 84.43±4.99 | 5.92±1.91 | 507.99±201.15 |
|  | LFN group | 4 | 61.12±11.76 | 3.68±1.37 | 381.10±82.19 |
|  |  | 6 | 58.59±21.22 | 3.5±2.45 | 327.02±93.44^*^ |
|  | SLF group | 4 | 63.51±5.07^**^ | 3.77±1.15 | 345.10±147.33^*^ |
|  |  | 6 | 44.49±12.63^**^ | 3.08±1.21 | 310.54±95.96^**^ |
| Pregnancy anemia model after IDA | Unpregnant group | 0 | 24.99±6.84^##^ | 0.93±0.25^##^ | 226.31±72.11^##^ |
|  | Physiological pregnancy group | 0 | 39.20±13.04^#^ | 1.64±0.59^#^ | 380.14±140.79^#^ |
|  | IDA pregnancy group | 0 | 64.68±18.74 | 2.81±0.86 | 598.68±130.03 |
|  | LFN group | 7 | 34.13±16.29^#^ | 1.50±0.44^##^ | 283.50±100.08^##^ |
|  | SLF group | 7 | 29.86±14.09^##^ | 1.17±0.61^##^ | 183.22±102.25^##^ |

Table 10 Effects of iron supplements on mRNA expression of liver iron metabolism related indicators in pregnant anemic rats

| Model | Group | Pre administration (week) | HAMP | FPN1 | TFR1 |
| --- | --- | --- | --- | --- | --- |
| Physiological pregnancy anemia model | Unpregnant group | 0 | 1.18±0.18^**^ | 0.90±0.11 | 0.58±0.16 |
|  | Physiological pregnancy group | 0 | 0.13±0.03 | 1.19±0.23 | 1.01±0.21 |
|  | LFN group | 4 | 0.29±0.09 | 1.03±0.14 | 0.68±0.26^*^ |
|  |  | 6 | 1.27±0.59^**^ | 1.66±0.41 | 0.82±0.44 |
|  | SLF group | 4 | 1.03±0.68^**^ | 1.10±0.55 | 0.41±0.30^**^ |
|  |  | 6 | 1.69±1.33^**^ | 1.43±0.64 | 0.76±0.81 |
| Pregnancy anemia model after IDA | Unpregnant group | 0 | 1.10±0.19^##^ | 0.49±0.28^#^ | 0.35±0.14^#^ |
|  | Physiological pregnancy group | 0 | 0.10±0.07 | 0.68±0.19 | 0.58±0.22 |
|  | IDA pregnancy group | 0 | 0.18±0.19 | 1.32±0.81 | 1.19±0.35 |
|  | LFN group | 7 | 0.24±0.24 | 0.85±0.54 | 0.77±0.43 |
|  | SLF group | 7 | 0.61±0.57 | 0.99±0.61 | 0.41±0.28 |

Note: Compared with the physiological pregnancy group, *,P＜0.05；**，P＜0.01；Compared with the IDA pregnancy group,#, P＜0.05.

Table 11 Effects of Iron Supplements on mRNA Expression of Iron Metabolism Related Indicators in the Small Intestine of Pregnant Anemia Rats

| Model | Group | Pre administration (week) | FPN1 | DMT1+IRE | DMT1-IRE | DMT1 |
| --- | --- | --- | --- | --- | --- | --- |
| Physiological pregnancy anemia model | Unpregnant group | 0 | 0.75±0.10* | 0.55±0.25* | 0.66±0.16 | 0.76±0.27 |
|  | Physiological pregnancy group | 0 | 1.77±0.33 | 1.65±0.39 | 0.85±0.48 | 1.70±0.45 |
|  | LFN group | 4 | 0.81±0.22* | 0.86±0.20 | 0.90±0.16 | 1.07±0.27 |
|  |  | 6 | 0.88±0.23* | 0.48±0.23* | 0.62±0.16 | 0.81±0.31 |
|  | SLFgroup | 4 | 0.67±0.32* | 0.53±0.28* | 0.82±0.25 | 0.81±0.24 |
|  |  | 6 | 1.12±0.63 | 0.62±0.20* | 0.74±0.16 | 0.79±0.37 |
| Pregnancy anemia model after IDA | Unpregnant group | 0 | 0.78±0.46^#^ | 0.53±0.25 | 0.49±0.27^##^ | 0.83±0.34^#^ |
|  | Physiological pregnancy group | 0 | 1.13±0.30 | 0.87±0.15 | 0.84±0.25 | 0.90±0.19^#^ |
|  | IDA pregnancy group | 0 | 1.31±0.63 | 1.27±0.64 | 1.17±0.67 | 1.67±0.78 |
|  | LFN group | 7 | 0.91±0.36 | 0.77±0.10 | 0.76±0.22 | 1.03±0.69 |
|  | SLFgroup | 7 | 1.10±0.45 | 0.91±0.18 | 0.78±0.26 | 0.95±0.74^#^ |

Note: Compared with the physiological pregnancy group, *,P＜0.05；**，P＜0.01；Compared with the IDA pregnancy group,#, P＜0.05.

Table 12 Effects of iron supplements on mRNA expression of iron metabolism related indicators in the placenta of pregnant anemic rats

| Model | Group | Pre administration (week) | FPN1 | DMT1 | TFR1 |
| --- | --- | --- | --- | --- | --- |
| Physiological pregnancy anemia model | Physiological pregnancy group | 0 | 1.16±0.17 | 1.18±0.21 | 1.14±0.25 |
|  | LFN group | 4 | 1.61±1.08 | 1.35±0.87 | 1.43±1.17 |
|  |  | 6 | 1.31±0.57 | 1.03±0.26 | 1.00±0.44 |
|  | SLFgroup | 4 | 1.30±0.36 | 1.16±0.61 | 1.15±0.27 |
|  |  | 6 | 1.04±0.22 | 0.81±0.25 | 0.88±0.26 |
| Pregnancy anemia model after IDA | Physiological pregnancy group | 0 | 1.17±0.35 | 1.17±0.35 | 0.73±0.33 |
|  | IDA pregnancy group | 0 | 1.48±0.64 | 1.48±0.64 | 1.23±0.86 |
|  | LFN group | 7 | 0.95±0.37^#^ | 0.95±0.37^#^ | 0.90±0.40 |
|  | SLFgroup | 7 | 0.95±0.34^#^ | 0.95±0.34^#^ | 0.68±0.38 |

Note: Compared with the physiological pregnancy group, *,P＜0.05；**，P＜0.01；Compared with the IDA pregnancy group,#, P＜0.05.
